# Molecular Dynamics for the Optimal Design of Functionalized Nanodevices to Target Folate Receptors on Tumor Cells

**DOI:** 10.1021/acsbiomaterials.3c00942

**Published:** 2023-10-13

**Authors:** Edoardo Donadoni, Giulia Frigerio, Paulo Siani, Stefano Motta, Jacopo Vertemara, Luca De Gioia, Laura Bonati, Cristiana Di Valentin

**Affiliations:** † Dipartimento di Scienza dei Materiali, Università di Milano-Bicocca, via R. Cozzi 55, 20125 Milano, Italy; ‡ Dipartimento di Scienze dell’Ambiente e del Territorio, Università di Milano-Bicocca, Piazza della Scienza 1, 20126 Milano, Italy; § Dipartimento di Biotecnologie e Bioscienze, Università di Milano-Bicocca, Piazza della Scienza 1, 20126 Milano, Italy; ∥ BioNanoMedicine Center NANOMIB, Università di Milano-Bicocca, via R. Follereau 3, 20854 Vedano al Lambro, Italy

**Keywords:** atomistic simulations, titanium dioxide, nanoparticles, folic acid, nanomedicine, tumor targeting

## Abstract

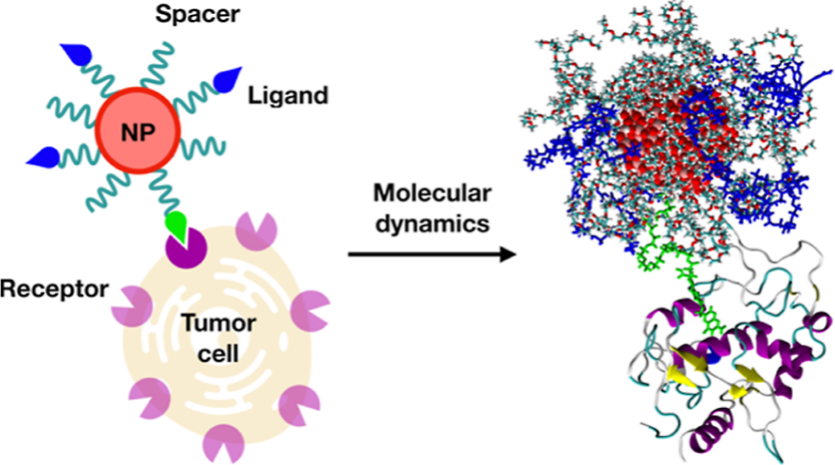

Atomistic details on the mechanism of targeting activity by biomedical nanodevices of specific receptors are still scarce in the literature, where mostly ligand/receptor pairs are modeled. Here, we use atomistic molecular dynamics (MD) simulations, free energy calculations, and machine learning approaches on the case study of spherical TiO_2_ nanoparticles (NPs) functionalized with folic acid (FA) as the targeting ligand of the folate receptor (FR). We consider different FA densities on the surface and different anchoring approaches, i.e., direct covalent bonding of FA γ-carboxylate or through polyethylene glycol spacers. By molecular docking, we first identify the lowest energy conformation of one FA inside the FR binding pocket from the X-ray crystal structure, which becomes the starting point of classical MD simulations in a realistic physiological environment. We estimate the binding free energy to be compared with the existing experimental data. Then, we increase complexity and go from the isolated FA to a nanosystem decorated with several FAs. Within the simulation time framework, we confirm the stability of the ligand–receptor interaction, even in the presence of the NP (with or without a spacer), and no significant modification of the protein secondary structure is observed. Our study highlights the crucial role played by the spacer, FA protonation state, and density, which are parameters that can be controlled during the nanodevice preparation step.

## 1 Introduction

With the birth of nanomedicine, new noninvasive protocols have been developed to defeat cancer. In this respect, strategies exploiting the active targeting of tumor cells have been successful and efficient. They consist of the molecular recognition between a ligand and its target, located on the surface of tumor cells. These cellular targets comprehend cell–surface carbohydrates, cellular antigens, and cell–surface receptors,^1^ which are overexpressed by cancer cells, such as the folate receptor (FR).^2,3^ The FR has a very strong and specific affinity with folic acid (FA), also known as vitamin B9, which makes FA an excellent targeting ligand for FR-targeted therapies.^4−6^

The FR family comprehends four homologous glycosylphosphatidylinositol (GPI)-anchored membrane proteins:^7^ FRα, located on the apical surface of healthy epithelial cells and overexpressed on tumor cells; FRβ, which is found on activated macrophages; and FRγ and FRδ, which are present, respectively, in bone marrow and T-cells and whose function remains unclear. For this reason, research has mainly focused on FRα. FRα is involved in the internalization of oxidized forms of folates that cannot be synthesized ex novo by the human body but need to be consumed in the diet. The FA internalization by healthy epithelial cells is carried out by the FRα proteins placed on their apical surface. Upon the cell tumorigenesis process, however, the disruption of cell junctions determines the loss of cell polarity, and consequently, the receptors become exposed to both apical and basal cell surfaces. The latter is in contact with the blood vessels; therefore, FRs find themselves exposed to folates from the bloodstream. This effect can be nicely exploited for an efficient targeting of tumors.^8^

The tertiary structure of FRα in complex with FA was resolved in 2013 by X-ray crystallography:^9^ the globular structure comprises 6 α-helices, 4 β-sheets, and many loop regions and is stabilized by 8 disulfide bonds formed by 16 conserved cysteine residues. The FA pteorate moiety is buried inside a hydrophobic binding pocket, where the FA amine group interacts with the FRα Asp81 residue: this is considered to be the key ligand–receptor interaction for the molecular recognition process. The glutamate portion of FA with its two carboxylic groups instead stays outside the binding pocket in a positively charged region of FRα.

Since then, much effort has been devoted to understanding the details of the molecular recognition of FA by FRα. In this respect, several theoretical studies, based on docking calculations and molecular dynamics (MD) simulations, have contributed to providing useful information on the FRα/FA interactions at an atomic resolution level. For instance, Gu and collaborators^10−13^ combined density functional theory (DFT), MD, molecular docking, and molecular mechanics/Poisson–Boltzmann surface area free energy calculations to perform a screening of several folate and antifolate derivatives and to determine how substitutional functional groups could enhance the ligand–receptor interaction. Moreover, Della-Longa and Arcovito set up metadynamics calculations and reported a free energy barrier for the FA escape from FRα of 18 kcal mol^–1^.^14^ Schaber et al., using unbiased classical MD instead, demonstrated that FA binding occurs spontaneously on the nanosecond time scale, without applying any bias to the system.^15^

Certainly, the striking interaction between FA and the FR can be exploited to design effective nanomedical devices for drug release or tumor imaging, which upon FA functionalization can be selectively driven toward FRα-expressing tumor cells. This allows to obtain a targeted therapy with limited side effects related to unwanted delivery of cargos at normal cells. In this context, FA has been successfully conjugated onto the surface of several carriers, either organic, including liposomes,^16^ dendrimers,^17^ polymeric micelles,^18^ and luteic acid/chlorin e6 aggregates,^19,20^ or inorganic nanoparticles (NPs).^21,22^ Among them, titanium dioxide (TiO_2_) NPs are used in the photodynamic therapy of tumors because of their excellent photocatalytic properties. In particular, the generation of reactive oxygen species upon UV–Vis light irradiation of TiO_2_ NPs^23−29^ leads to the oxidation of cell membrane’s lipids and, at the end, to the disruption of the tumor cells.

To the best of our knowledge, no atomistic simulations exist that tackle the interaction of FA with FRα when the ligand is conjugated to a NP. In a recent previous work by some of us,^30^ we designed models of FA-functionalized nanodevices based on bare or polyethylene glycol (PEG)-coated (for enhanced stealth properties) TiO_2_ spherical NPs and investigated their stability and behavior in water or physiological environment.

Here, we use classical MD simulations, free energy calculations, and machine learning approaches to assess the capabilities of these nanomedical devices to target FRα and determine the interaction mechanism. In particular, through molecular docking, we identified the lowest energy conformations of FA inside the FRα binding pocket in its experimentally obtained crystal structure when complexed with FA.^9^ Starting from the best FA docking poses, we set up classical MD simulations of FRα bonded to a single FA ligand in a realistic physiological environment and estimated the binding free energy that can be compared to the existing experimental data.^9^ Then, we introduced the presence of the spherical TiO_2_ NP, which we decorated with increasing density of FAs, in both their neutral and monodeprotonated states and which are either directly covalently bonded to the surface or through PEG spacers. Within the simulation time framework, we confirmed the stability of the ligand–receptor interaction, even in the presence of the NP (with or without a spacer). Moreover, thanks to the atomic resolution provided by the level of theory of this study, we could also provide details of the structural and dynamical interplay among the different components of the nanoconjugate with FR, i.e., the photoactive TiO_2_ core, the PEG polymer coating, and the targeting FA molecules, and prove the crucial role played by the FA density and protonation state on the interaction of the functionalized nanodevice with the target protein.

## 2 Computational Details

### 2.1 Nomenclature

In this section, we introduce the nomenclature used along the manuscript to indicate the various systems considered in this work. In the absence of the NP, the FA molecule docked inside the FR binding pocket is labeled LIG, with the protonation state indicated at the apex, i.e., LIG^0^, LIG^1–^, or LIG^2–^ when it is neutral, singly, or doubly deprotonated, respectively. Referring to LIG at different protonation states, we use multiple apexes, such as “LIG^0,1–^” or “LIG^0,1–,2–^”. Finally, for the receptor/ligand complexes, we labeled FR/LIG^0^, FR/LIG^1–^, and FR/LIG^2–^, where the symbol “/” represents a noncovalent interaction.

The FA molecules attached to the NP are labeled FAs^b1–^ or FAs^b0^, depending on whether they are deprotonated or not, except for the one FA molecule in the FR pocket that is labeled LIG^b1–^ or LIG^b0^, where *b* stands for covalently bonded to the NP (either through a PEG chain or directly to the NP surface). Accordingly, the overall FA-functionalized PEGylated nanosystems are denoted as TiO_2_–PEG-*n*-FAs^b0^ or TiO_2_–PEG-*n*-FAs^b1–^, where *n* indicates the number of FA molecules attached to the PEG chains (either 10 or 20), and upon interaction with FR become FR/TiO_2_–PEG-*n*-FAs^b0^ and FR/TiO_2_–PEG-*n*-FAs^b1–^. Here, “/” and “-” stand for noncovalent interactions and covalent bonding, respectively. Finally, the non-PEGylated FA-functionalized systems are named TiO_2_-48-FAs^b0^-γ and TiO_2_-48-FAs^b1–^-γ, where γ means that all of the FA molecules (48) are covalently bonded to the TiO_2_ surface through their γ-COOH group. Upon interaction with FR, they become FR/TiO_2_-48-FAs^b0^-γ and FR/TiO_2_-48-FAs^b1–^-γ, respectively.

### 2.2 Rigid Receptor Docking Calculations

System preparation and docking calculations were performed through the Glide package^31^ of the Schrödinger Suite program (release 2021-1). The protein structure, obtained by X-ray diffraction (PDB ID: 4LRH, 2.8 Å resolution), was prepared using the Protein Preparation Wizard.^32^ In this step, the protonation states of FRα (hereinafter FR) amino acids (AAs) at pH = 7.0 were assigned. The ligand molecule (referred to as LIG) was prepared with the LigPrep tool. We considered the lactamic tautomeric form of FA since a combined NMR and DFT study demonstrated that it is the most stable FA tautomeric form in solution.^33^ We studied three cases, the first where LIG is neutral (LIG^0^) and the other where one (LIG^1–^) or both of the two (LIG^2–^) carboxyl groups of LIG glutamate are deprotonated. In the case of LIG^1–^, the dissociated carboxyl group is α-COOH, as it is the most acidic out of the two.^30^ The motivation to test different protonation states comes from the fact that all three of them, in principle, could be stabilized in the FR binding pocket, where the surrounding environment is different from the solution at physiological pH. Rigid receptor docking was performed with the Glide software^34^ and the OPLS_2005 force field (FF).^35^ The grid box was defined by using the ligand crystallized in complex with the protein. We used the glide extra precision (XP) scoring function to rank the computed docking poses.

The LIG binding energy, Δ*E*_binding_, was defined as shown in eq 1
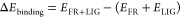
1where *E*_FR+LIG_, *E*_FR_, and *E*_LIG_ are, respectively, the total energy of the complex, isolated receptor, and isolated ligand. The binding energy calculations were performed through the MacroModel program implemented in the Maestro software, using the OPLS_2005 FF, in generalized born implicit solvent (GBIS).^36^

### 2.3 Classical MD Simulations

The MD simulations of LIG in complex with FR that did not involve the TiO_2_ NP were carried out by employing the NAMD 2.13 open-source code^37^ and its CUDA module for GPU-accelerated calculations, where FR and LIG were described, respectively, by the CHARMM36^38^ and the CGenFF^39^ FFs. The best docking poses for the FR/LIG complex (Section 2.2) were chosen as starting point geometries. The FR/LIG systems were immersed in a 100 × 100 × 100 Å^3^ cubic CHARMM-modified rigid TIP3P^40^ water box in the presence of 0.15 M NaCl with the CHARMM-GUI interface.^41^ The FR net charge (+3) was neutralized with 3, 2, or 1 additional Cl^–^ ions, in the case of LIG^0^, LIG^1–^, and LIG^2–^, respectively, randomly put in the bulk water phase. Initial structures were relaxed with the Conjugate Gradient algorithm and then equilibrated at a temperature of 303 K, held constant by using a Langevin friction force with a damping coefficient of 0.1 ps^–1^ (NVT ensemble), under periodic boundary conditions, making use of a constant 2 fs time step for the integration of the Newton’s equations of motion, by means of the impulse-based Verlet-I/r-RESPA integrator.^42^ The particle mesh Ewald (PME) scheme^43^ was used to evaluate the long-range electrostatic interactions using a real-space cutoff of 12 Å. Lennard-Jones interactions were truncated with a 12 Å cutoff, with a switching function applied between 10 and 12 Å. SHAKE/RATTLE^44,45^ and SETTLE^46^ algorithms in combination were used to impose holonomic constraints on all covalent bonds including hydrogen atoms. During the 1 ns equilibration phase, a constraint on the initial geometry was imposed on the FR backbone atoms. The constraints were then removed along the following production phase, performed at 303 K in an NPT ensemble, where a Nosé–Hoover Langevin piston scheme^47,48^ with a piston period of 50 fs and a piston decay of 25 fs ensured constant pressure (1 atm). During MD production, the phase space was explored for a total simulation time of 500 ns.

For the MD simulations involving the TiO_2_ NP, we made use of the LAMMPS (29 Sep 2021 version^49^) open-source code. The bare TiO_2_ NP model was designed by our group in previous works^50,51^ and consists of a spherical anatase NP, carved from the crystalline bulk anatase structure and fully relaxed, first at the DFTB level of theory with a simulated annealing procedure, followed by a DFT-B3LYP optimization. The stoichiometry of the NP is (TiO_2_)_223_·10H_2_O, and it is characterized by an approximate diameter of 2.2 nm. The surface 5- or 6-fold coordinated Ti atoms of the NP were saturated, in a recent paper by some of us,^30^ by 48 FA molecules, making either bidentate or chelated coordinative bonds upon dissociation of the FA γ-COOH group (TiO_2_-48-FAs^b^-γ). The PEGylated NP model was built and validated by our group in previous studies,^52,53^ where the NP was grafted with 50 methoxy-PEG molecules (H_3_C–[OCH_2_CH_2_]_*n*_–OH, with *n* = 11) and fully optimized through atomic relaxation at the DFTB level of theory and MD simulations. On this model, we added either 10 or 20 FA molecules, as randomly distributed as possible, by a covalent linkage through an ester bond involving the terminal C of PEG methyl and the FA γ-carboxylic group (TiO_2_–PEG-*n*-FAs^b^). The FA-functionalized PEGylated TiO_2_ systems were only partially relaxed at the DFTB level of theory.^30^

The NP was described by an improved Matsui-Akaogi FF, reparametrized by Brandt and Lyubartsev,^54^ while CGenFF was chosen for the adsorbed FA, PEG, or PEG-FA molecules. The FF used for the functionalized NP has been tested and validated for a TiO_2_ NP tethered with small organic molecules by some of us^55^ and employed in previous works.^30,56^ The FR/TiO_2_–PEG-*n*-FAs^b^ and FR/TiO_2_-48-FAs^b^-γ topologies were generated by means of the Moltemplate^57^ package for LAMMPS, and the systems were immersed, respectively, in a 170 × 170 × 170 Å^3^ or 135 × 135 × 135 Å^3^ cubic rigid CHARMM-modified TIP3P water box, built up with the Packmol^58^ software. The starting point configuration of FR in complex with the LIG^b^ molecule is the last snapshot of the 500 ns MD simulation of the corresponding system without the NP. During all the atomistic MD simulations, we held fixed the geometry of the NP core and of the anchoring PEG –OH or FA –COO^–^ groups at the DFTB geometry, and we treated the NP as a rigid body, free to translate and rotate as a whole, fixing its internal degrees of freedom at the DFTB-optimized geometry through the RIGID package^59^ in LAMMPS, as we did in some previous works by some of us.^30,56,60^ This approach keeps the DFTB relative atomic positions within the TiO_2_ NP and avoids any core misshaping during the MD simulation. Whatever was not fixed, instead, was free to evolve in time, at 303 K (NVT ensemble), making use of a constant 2 fs time step for the integration of Newton’s equations of motion, where the SHAKE algorithm imposed holonomic constraints on all the covalent bonds involving hydrogen atoms. Periodic boundary conditions were used. Long-range electrostatic interactions were evaluated by the particle–particle particle–mesh solver using a real-space cutoff of 12 Å. Short-range Lennard-Jones interactions were smoothly truncated with a 12 Å cutoff by means of a switching function applied between 10 and 12 Å. Several minimization steps ensured that no overlaps between the atoms occurred, then a 1 ns equilibration followed, where constraints were applied to the FR backbone, and finally, after removing the constraints, the phase space was explored for a total production time of 200 ns.

### 2.4 Simulation Analysis

Atomic radial distribution function (rdf) was computed with the Radial Distribution Function plugin of VMD,^61^ considering all the atoms at a given distance *r* from the reference and falling in a spherical shell with a thickness of 0.1 Å.

The hydrogen bond analysis was carried out by means of the Hydrogen Bonds tool provided by VMD. The criteria for the H-bonds classification were set as 3.0 Å for the maximum donor–acceptor distance and 20° for the maximum acceptor–donor–hydrogen angle.

The calculations of the nonbonded interaction energies (vdW and electrostatic) were performed through the USER-TALLY package, implemented in the LAMMPS code and with the NAMD Energy tool for VMD.

Contact surface area computation was conducted exploiting the Voronoi tessellation method as implemented in the VORONOI^62^ package of LAMMPS.

All distances and angles were measured with the CPPTRAJ^63^ module implemented in AmberTools and with the open-source software LOOS 3.1.^64^ The angle vs distance heatmaps were plotted with an in-house Python script.

Protein secondary structure analysis was conducted with the Timeline utility of VMD.

#### 2.4.1 Free Energy Calculations

The simulation protocol used in this work for the free energy calculations involves two steps: the first step is Steered Molecular Dynamics (SMD), followed by Umbrella Sampling (US) simulations. The SMD step was necessary to collect a series of configurations along the selected reaction coordinate (ξ) that represents the unbinding of the complex. During this procedure, the subunits were pushed away from one another by applying a biasing potential to their centers of mass (COMs). The COMs of the subunits were oriented along the *x*-direction of the Cartesian space. During the pulling simulations, a harmonic potential of 1000 kJ mol^–1^ nm^–2^ was applied only along the *x*-direction with a pull rate of 0.01 nm ps^–1^. The *x*-dimension of each simulation box was enlarged to guarantee that the pull distance was always less than half of the length of the box vector along which the pulling was conducted. Frames representing a COM spacing of 0.1 nm, referred to as configurations, were extracted from the pulling trajectories and were used as starting points for the US simulations. The US simulations were performed at 303 K and 1 atm for 2 ns, where each configuration was restrained within a window corresponding to the chosen COM distance by applying a harmonic potential of 1000 kJ mol^–1^ nm^–2^. All the SMD and the US simulations were carried out using the CHARMM36 FF and the GROMACS^65^ code. The Potential of Mean Force (PMF) was calculated from the umbrella histograms using the Weighted Histogram Analysis Method (WHAM)^66^ implemented in GROMACS. For the PMF calculations, the following parameters were used: 50 bins per 1 nm, 100 bootstraps to obtain an average bootstrapped PMF, and error estimates. The PMF was set to zero at the protein–ligand COM distance, where the pull force dropped to the minimum. Individual umbrella histograms were weighted with estimated integrated autocorrelation times (IACTs) smoothed along the reaction coordinate (*x*-direction) using Gaussian functions with standard deviation σ = 0.15 nm.^67^

#### 2.4.2 Cluster Analysis with Self-Organizing Maps

A Self-Organizing Map (SOM) is an unsupervised learning method that allows the visualization of multidimensional data in a low-dimensional representation.^68^ Several applications of SOMs to the analysis of biomolecular simulations can be found in the literature ranging from clustering of ligand poses in virtual screening^69^ to analysis of pathways in enhanced sampling MD simulations.^70−73^

In this work, we used the PathDetect-SOM tool^74^ for the SOM training using a 10 × 10 sheet-shaped SOM (without periodicity across the boundaries) with a hexagonal lattice shape. The input features used to train the SOM consisted of a set of protein–ligand intermolecular distances, defined by visual inspection of the MD simulation. This set of distances was used to build the set of conformations for SOM training.

The training algorithm for SOMs involves several steps:

1. Initialization: at the outset, a grid of neurons arranged in a two-dimensional lattice is created. The grid’s dimensions are determined by a specified hyperparameter. Each neuron is assigned an initial weight vector, randomly initialized.
2. Data sampling: during each training iteration, a data point is randomly selected from the data set. This data point serves as the input for the current iteration.
3. Competition: the algorithm calculates the similarity between the input data point and the weight vectors of all neurons in the grid. This similarity measurement, based on Euclidean distance, identifies the Best Matching Unit (BMU). The BMU is the neuron whose weight vector is most similar to the input data point.
4. Cooperation: a neighborhood Gaussian function is employed. This function defines the extent to which the BMU influences its neighboring neurons. Neurons in closer proximity to the BMU undergo more substantial weight updates, while those farther away are adjusted to a lesser degree.
5. Learning rate decay: to facilitate convergence, the learning rate (controlling the magnitude of weight updates) and the radius of the neighborhood function decrease as training progresses. This enables more refined adjustments in later stages of training.
6. Iteration: the training process continues iteratively by repeatedly sampling data points, updating neuron weights, and adjusting the radius of the neighborhood function over 5000 training cycles.

After training, each frame of the simulations was assigned to a neuron on the map, and each neuron represented a protein–ligand conformational microstate. In a second step, the neurons were further grouped into a small but representative number of clusters by agglomerative hierarchical clustering using Euclidean distance and complete linkage. The number of clusters was chosen based on the Silhouette profile.

## 3 Results and Discussion

The presentation of the results is organized as follows: in Section 3.1, we describe the docking calculations carried out to find the most stable conformations of LIG at different protonation states inside the protein binding pocket of the crystal structure of FRα in complex with LIG. These docking poses are then used as the starting point for the atomistic MD simulations of the ligand–receptor systems in solution at physiological salt concentration in Section 3.2. After that, in Section 3.3, we analyze the effect of the presence of a conjugated TiO_2_ NP on the ligand–FR interactions, again considering different protonation states of both LIG^b^ and FAs^b^.

### 3.1 Docking of One FA Molecule Inside the FR Binding Pocket

As a preliminary computationally cheap investigation, we set up docking calculations to determine the best poses (i.e., conformations) of LIG inside the FR binding pocket. The crystal structure of FRα in complex with FA is present in the Protein Data Bank (PDB code 4LRH).^9^ Since no information is provided on the position of the hydrogen atoms (which are not detected by X-rays), we processed the file with the Maestro software to set the correct protonation state of the AAs at physiological pH based on the pK_a_ values of their titratable groups. Regarding the ligand, we considered three protonation states: LIG^0^ (neutral), LIG^1–^, or LIG^2–^ (with one or two LIG carboxyl groups deprotonated, respectively). This choice was made to determine which LIG protonation state establishes the strongest ligand–protein interaction since all three of them, in principle, could be stabilized in the FR binding pocket, where the surrounding environment is different from the solution at physiological pH. In the case of LIG^1–^, the dissociated carboxyl group is α-COOH (i.e., the –COOH bonded to the C atom in α position with respect to the glutamate amide C atom), as it is the most acidic of the two types of LIG carboxylic groups.^75^ We performed rigid receptor docking calculations where the FR geometry was kept fixed at the crystallographic position, while LIG was free to move and rearrange inside the FR binding site.

**Figure 1:**
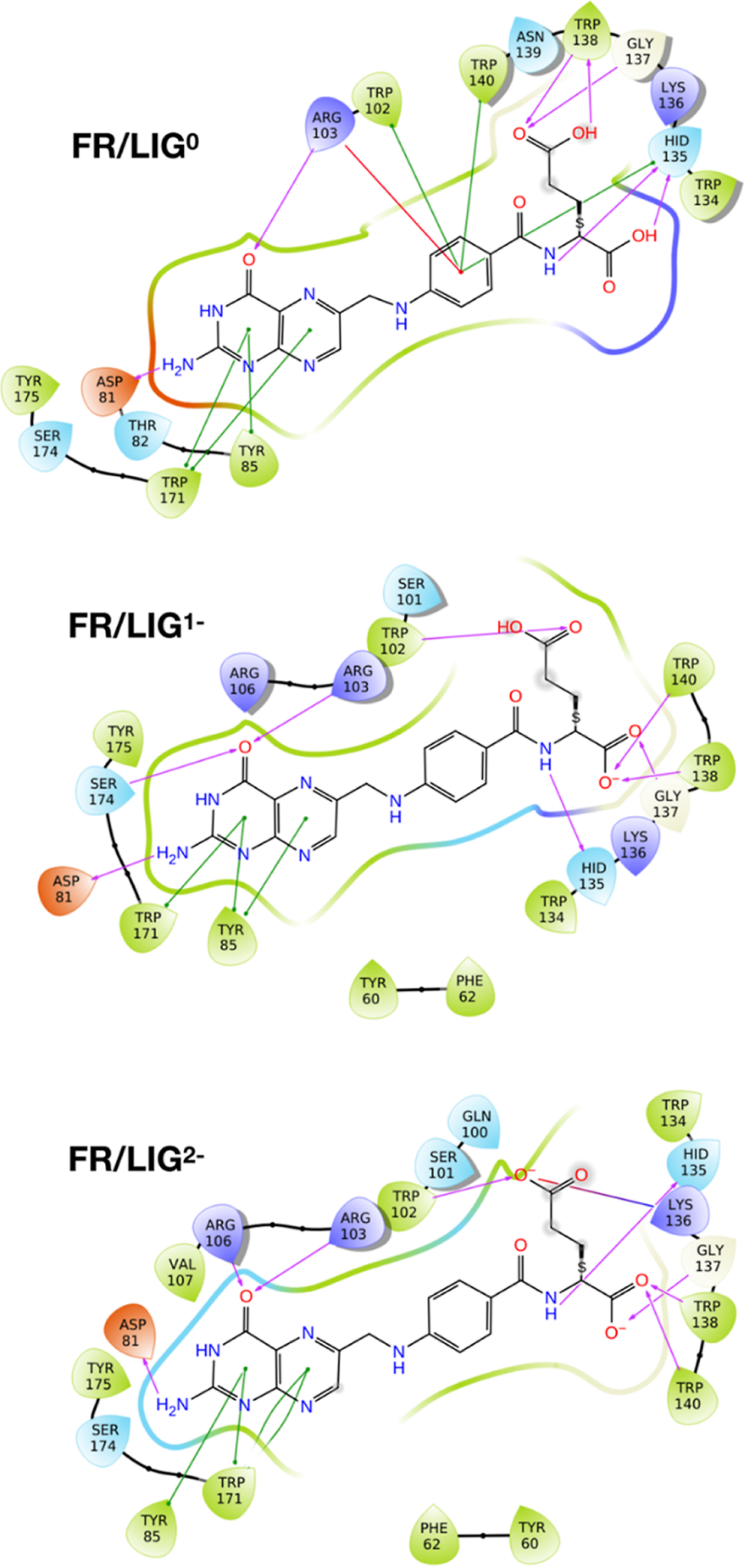
Interaction diagrams of LIG^0,1–,2–^ in the FR binding pocket from docking calculations. Green and red lines indicate the π–π and the cation–π stacking interactions, respectively, whereas violet arrows represent the hydrogen bonds.

Figure 1 shows the interaction diagrams of LIG^0,1–,2–^ inside the FR binding pocket relative to the best LIG poses: (i) the LIG pterin fragment is docked inside the FR binding pocket, where its amine group forms one H-bond with the Asp81 residue, which is considered to be the most important ligand–receptor interaction for the molecular recognition process.^9^ In addition, the pterin aromatic rings for all LIG^0,1–,2–^ interact with the Trp171 and Tyr85 residues through π–π stacking interactions, and the pterin carbonyl oxygen is an H-bond acceptor from Arg103 (LIG^0,1–,2–^) and Ser174 (LIG^1–^) and Arg106 (LIG^2–^); (ii) for the LIG glutamate, α-COOH is H-bonded to Gly137, Trp138, and Trp140 residues in LIG^1–,2–^, γ-COOH to Trp102 in LIG^1–,2–^, and Lys136 in LIG^2–^, whereas the N atom to Hid135 in LIG^1–,2–^, while in LIG^0^, the benzene ring forms π–π stacking interactions with Trp102, Hid135, and Trp140 residues and cation–π stacking with Arg103.

These results are in good agreement with X-ray crystallography,^9^ except for the fact that LIG pterin is experimentally found to also interact with Hid135 and not with Tyr85. In particular, the glutamate fragment interactions are in full agreement when LIG is doubly deprotonated (LIG^2–^). Also, the experimental observation that the *p*-aminobenzoate group does not establish any interaction with the protein would cause the exclusion of LIG^0^. We have also calculated the Root-Mean-Square Deviation (RMSD) of atomic positions of FA docking poses with respect to the crystallographic structure: 2.417, 0.914, and 1.070 Å for LIG^0^, LIG^1–^, and LIG^2–^, respectively. The RMSD is excellent for LIG^1–^ and LIG^2–^, while it is slightly higher for LIG^0^, but the differences arise from the rotation of the FA glutamate, which is more exposed to the solvent and establishes fewer interactions with the protein. Based on this comparative analysis, we can conclude that docking poses are proper starting points for the MD simulations of the FR/LIG^0,1–,2–^ systems, and we are led to believe that LIG is probably in a deprotonated form.

On the best docking poses, we calculated the LIG binding energy to FR (Δ*E*_binding_, eq 1) in a GBIS implicit water solvent using the OPLS_2005 FF. The results are presented in Table 1.

**Table 1:** LIG^0,1–,2–^ Binding Energies in the FR Binding Pocket from Docking Calculations in the GBIS Implicit Water Solvent

System	Δ*E*_binding_ (kcal mol^–^^1^)
FR/LIG^0^	–34.418
FR/LIG^1^^–^	–49.369
FR/LIG^2^^–^	–56.586

We observe that the binding energy gets more negative as the ligand negative charge increases: these results are rationalized considering that the FR AAs within 5 Å from the ligand bear a total net charge of +3; therefore, the trend of FR-LIG electrostatic interaction is LIG^2–^ > LIG^1–^ > LIG^0^, see Table S1.

### 3.2 MD Simulations of One FA Molecule in the FR Pocket at Physiological Conditions

**Figure 2:**
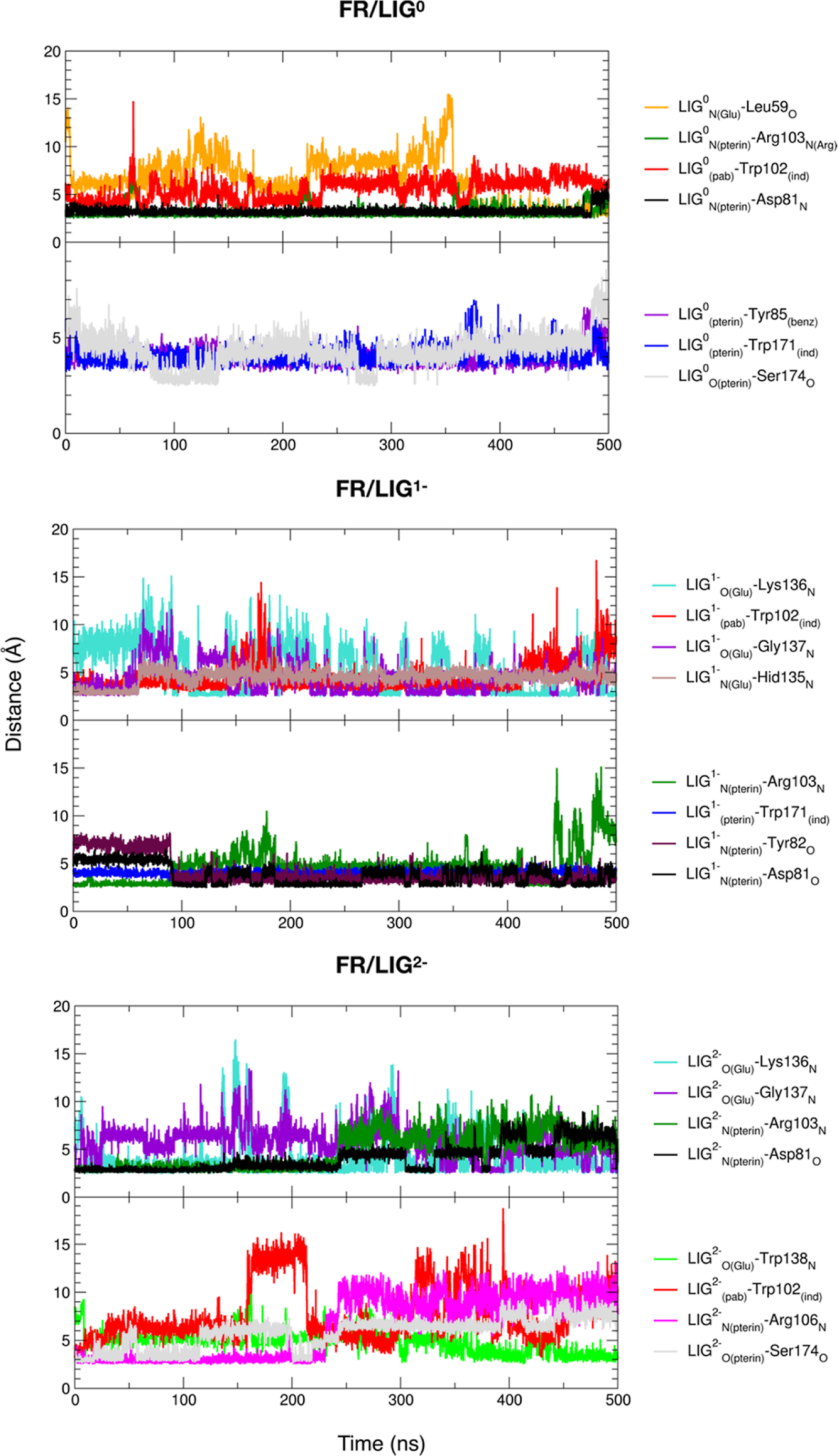
Time evolution of distances between selected atoms of LIG^0,1–,2–^ and FR AAs for the 500 ns simulations of the FR/LIG^0,1–,2–^ systems in a physiological environment.

Based on the results from the previous section, here we discuss the 500 ns atomistic MD simulations of FR in complex with LIG at different ligand protonation states in a physiological environment, where the docked structures are the starting point geometries for the simulations. In Figure 2, the time evolution of distances between selected atoms of LIG and FR AAs of the active site, along the 500 ns MD simulations, is reported for the FR/LIG^0,1–,2–^ systems.

In general, although distance values oscillate with time, it is important to underline that the ligand is found inside the FR binding pocket during all the three 500 ns MD simulations. Moreover, a large percentage of ligand–receptor interactions found in the crystal structure^9^ and from our docking calculations (Section 3.1) are also observed along the MD simulations, especially in the case of FR/LIG^2–^. Only the interaction of LIG glutamate with Trp140 is not observed in any of the three simulations, which may be due to the fact that the carboxyl groups of LIG are exposed to the solvent; therefore, there is a competition between ligand–protein and ligand–solvent interactions.

A conformational analysis of FR allows us to determine whether the protein secondary structure is modified along the 500 ns MD simulations. According to the results in Figure S1, all the main secondary structure elements (i.e., α-helices and β-sheets) are preserved all over the simulation; therefore, we can state that the FR crystal structure conformation is maintained, when immersed in a saline solution of 0.15 M NaCl.

In Table S2, the interplay among ligand, receptor, and solvent is presented in terms of intermolecular H-bonds and nonbonded interaction energies, calculated as an average of the last 100 ns of each simulation. In particular, as one would expect, the number of H-bonds between LIG and FR or between LIG and water increases with the negative net charge of the ligand from LIG^0^ to LIG^1–^ to LIG^2–^. In parallel, the ligand–receptor electrostatic interactions and the ligand solvation energy grow. For a more detailed analysis, see the text below Table S2.

**Figure 3:**
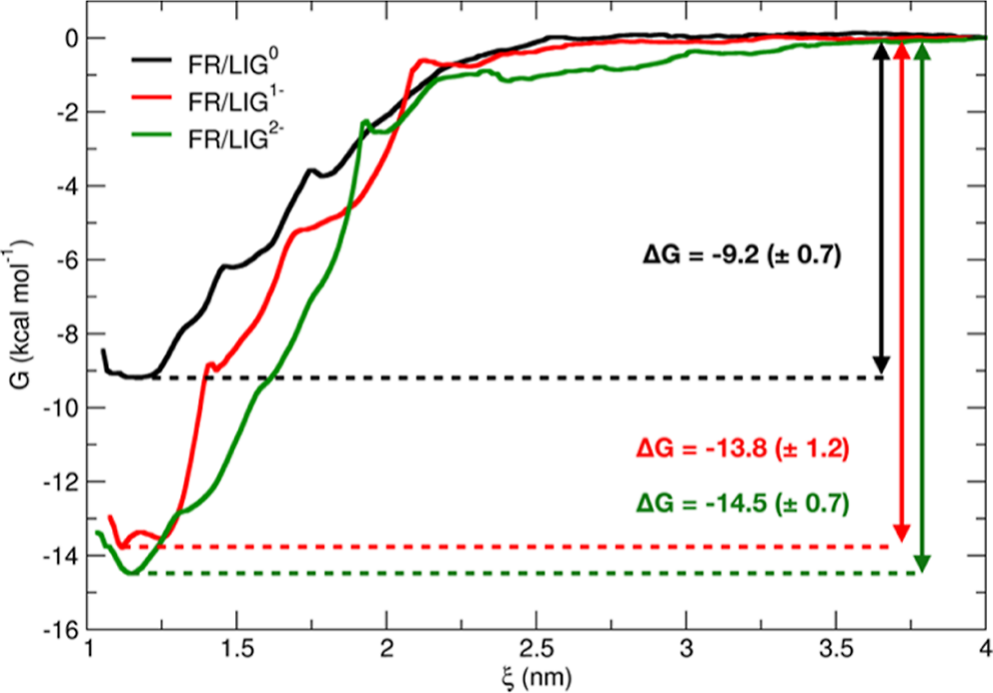
Free energy profiles of LIG binding to FR calculated from US simulations for FR/LIG^0^ (black), FR/LIG^1–^ (red), and FR/LIG^2–^ (green) systems.

A more quantitative analysis of the ligand–receptor interaction is based on thermodynamic data, namely, the Gibbs binding free energy of LIG inside the FR binding pocket (see Section 2.4.1 for computational details). The PMF profiles of LIG binding to FR at the three LIG protonation states were calculated from the umbrella histograms (Figures S2–S4) using the WHAM, and they are reported in Figure 3. We clearly observe a greater affinity of FR for the deprotonated forms of LIG (red and green lines) with respect to the protonated state (black line). Moreover, the results suggest that the binding is slightly stronger for LIG^2–^ than LIG^1–^: however, the difference in the binding free energy values lies within the estimated error bar, and therefore, it is not significant. Our results are consistent both with the experimental finding by Chen et al.,^9^ who reported a FA dissociation constant of 200 pM, corresponding to a dissociation free energy value of about 14 kcal mol^–1^, and with the metadynamics calculations by Della-Longa and Arcovito,^14^ who obtained a dissociation free energy value of about 18 kcal mol^–1^. This comparison between experimental and computational data suggests that the most probable protonation state of LIG inside the FR binding pocket is either LIG^1–^ or LIG^2–^.

**Figure 4:**
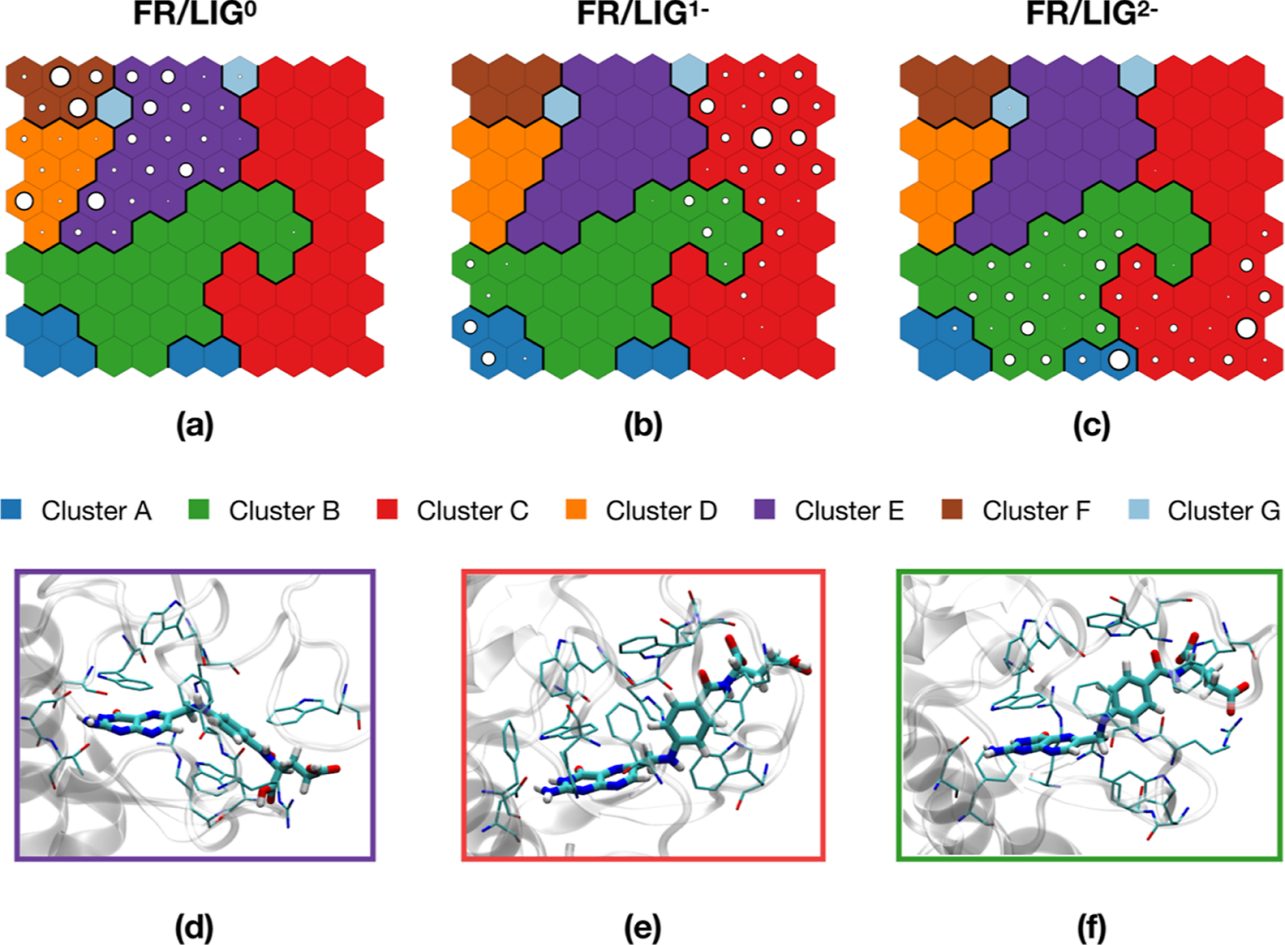
Results of the SOM/cluster analysis using the FR/LIG^0^, FR/LIG^1–^, and FR/LIG^2–^ 500 ns trajectories as a training set: neuron population map of FR/LIG^0^ (a), FR/LIG^1–^ (b), and FR/LIG^2–^ (c) systems; graphical representation of ligand conformation inside the FR binding pocket for the representative neuron of the most populated cluster of FR/LIG^0^ (d), FR/LIG^1–^ (e), and FR/LIG^2–^ (f) systems.

SOMs (see Section 2.4.2 for analysis details) were used to perform cluster analysis on the conformations of FA inside the FR binding pocket. We trained a 10 × 10 sheet-shaped SOM (without periodicity across the boundaries) using a data set of intermolecular distances between protein and ligand, as shown in Figure S5a. Figure 4 presents the results of the cluster analysis using the 500 ns trajectories of the FR/LIG^0^, FR/LIG^1–^, and FR/LIG^2–^ systems as a training set. Specifically, we depict the respective neuron population map (Figure 4a–c), where the dimension of the white circles represents the population size of each neuron cluster. The seven clusters are defined by the algorithm based on agglomerative hierarchical clustering using Euclidean distance and complete linkage, and they are labeled with a capital letter in alphabetical order (from A to G). The optimal number of clusters (7) was chosen as the one with the highest silhouette score in the range 5–10 (Figure S5b).

Additionally, we provide a graphical representation of the ligand conformation inside the FR binding pocket for the representative neuron of the most populated cluster in each protonation state (Figure 4d–f).

All these three clusters (Figure 4d–f) exhibit the same binding mode of the pterin ring, primarily influenced by the interaction between the ligand amine group and Asp81, as well as the stacking interactions with the Tyr85 and Trp171 rings. Differently, the region of the ligand containing the carboxylic acid groups exhibits the highest variance during the simulations. This last observation is partially expected because, despite their interactions with specific protein residues (e.g., Lys136, Trp140, and Trp138), the carboxylic groups reside in a solvent-exposed region, granting them increased mobility and contributing to the higher variance observed during the simulations.

The analysis of the population revealed that the simulations of LIG^1–^ and LIG^2–^ are more similar to each other. Indeed, even if they do not map the same exact neurons, their frames are assigned to the same clusters: for instance, clusters A, B, and C are populated by neurons coming from both FR/LIG^1–^ and FR/LIG^2–^ trajectories (Figure 4b,c), whereas the FR/LIG^0^ system occupies clusters D, E, and F (Figure 4a). This is due to a loss of interactions with Lys136, Trp140, and Trp138 by the carboxylic groups and a consequent shift toward Arg61.

To conclude this section on SOMs, based on the results, we confirm that the protonation state of the second carboxyl group has a lesser impact on the energetic stability of the binding modes compared to the deprotonation of the first carboxylic acid group, and this is in agreement with what was obtained from the free energy profiles in Figure 3. In fact, we do not observe relevant differences in the ligand binding geometry of the two deprotonated systems (Figure 4e,f).

### 3.3 Effect of the NP: MD Simulations of TiO_2_–PEG-*n*-FAs^b^ and TiO_2_-48-FAs^b^-γ in the FR Pocket at Physiological Conditions

In this section, we report the results obtained studying the dynamics of PEGylated or non-PEGylated, FA-functionalized TiO_2_ NPs where one of the FA molecules is docked inside the FR binding pocket in either its neutral (LIG^b0^), described in Section 3.3.1, or singly deprotonated state (LIG^b1–^), described in Section 3.3.2. In the presence of the NP, the doubly deprotonated state (LIG^b2–^) cannot even be conceived because the γ–COOH groups are used as the anchoring groups, either through direct covalent bonds to the surface Ti atoms or through ester bonds to the PEG chains.

The functionalized NP systems considered here are some of those modeled in our previous work:^30^ (i) TiO_2_–PEG-10-FAs^b^ and (ii) TiO_2_–PEG-20-FAs^b^, where 50 methoxy-PEG_500_ chains are grafted onto the NP surface and, respectively, 10 and 20 of them are covalently conjugated to FA through an ester bond involving the FA γ-COOH group, and (iii) TiO_2_-48-FAs^b^-γ, where 48 FA molecules have their γ-COOH groups dissociated and bound (either in a bidentate or chelated mode) to surface Ti atoms. In all cases, the starting point configuration of the FR in complex with the LIG^b^ molecule is the last snapshot of the 500 ns MD simulation of the corresponding FR/LIG system (Section 3.2). All the 200 ns simulations were performed at 303 K in a 0.15 M NaCl water solution after a 1 ns equilibration phase (see computational details in Section 2.3). For nomenclature purposes, hereinafter the FA molecule inside the FR pocket will be referred to as LIG^b0^ (neutral) or LIG^b1–^ (deprotonated) in order to distinguish it from the other undocked FA molecules (referred to as FAs^b0^ or FAs^b1–^).

#### 3.3.1 FAs in Their Neutral State

**Figure 5:**
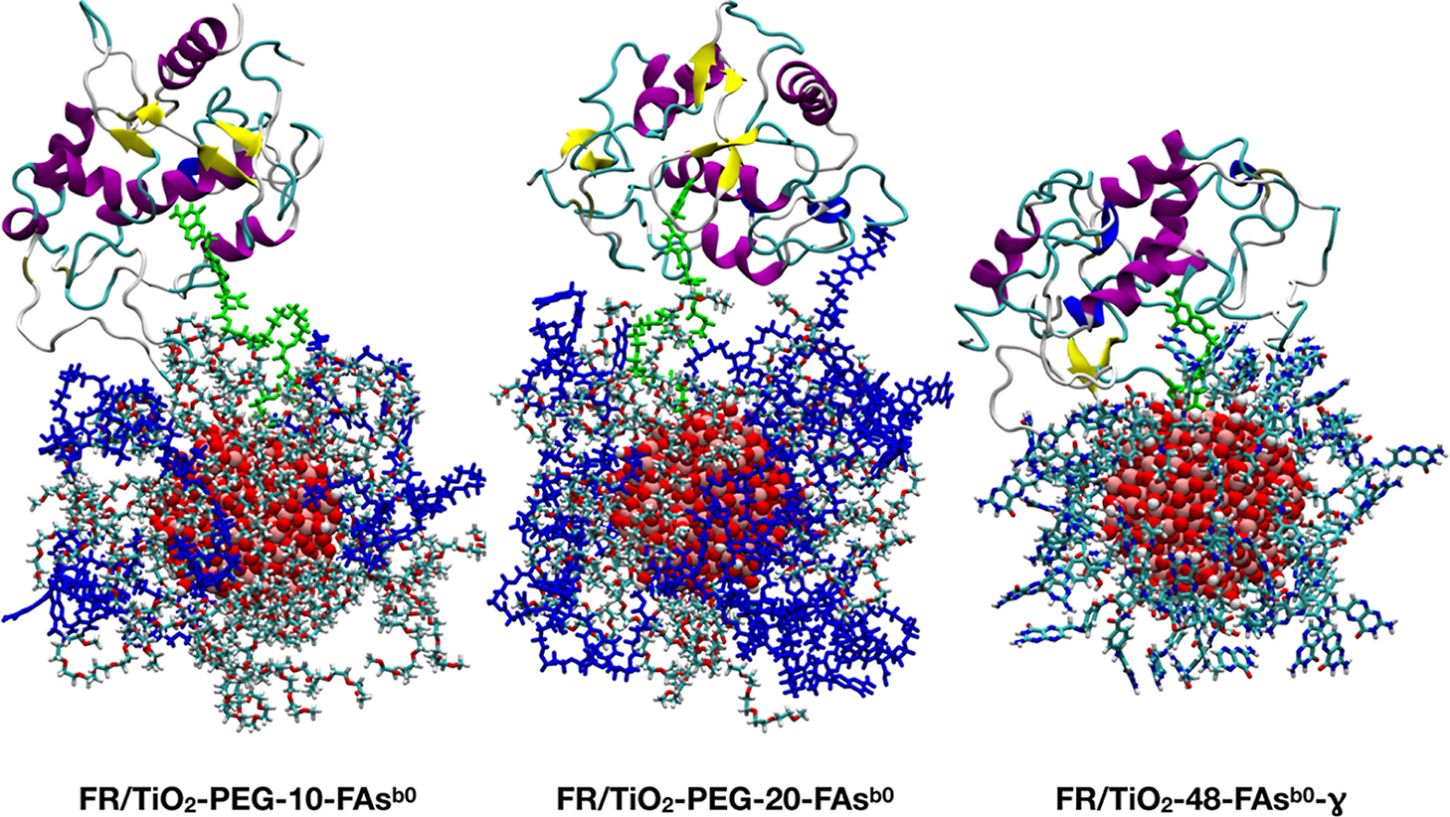
Last frame snapshots from the 200 ns MD simulations of the FR/TiO_2_–PEG-10-FAs^b0^, FR/TiO_2_–PEG-20-FAs^b0^, and FR/TiO_2_-48-FAs^b0^-γ systems. Titanium is shown in pink, oxygen in red, carbon in cyan, nitrogen in blue, and hydrogen in white. LIG^b0^ and PEG-LIG^b0^ chains are displayed in green, and FR is indicated in cartoon representation which is colored according to its secondary structure. In the PEGylated systems, the PEG-LIG^b0^ chains are represented in blue.

In Figure 5, we report the last frame snapshots from the 200 ns MD simulations of the FR/TiO_2_–PEG-10-FAs^b0^, FR/TiO_2_–PEG-20-FAs^b0^, and FR/TiO_2_-48-FAs^b0^-γ systems. We first observe that LIG^b0^ is still docked inside the FR binding pocket at the end of all of the simulations. In addition, in FR/TiO_2_–PEG-20-FAs^b0^, FR also interacts with one undocked FA^b0^ molecule and seems to be closer to the functionalized NP than in FR/TiO_2_–PEG-10-FAs^b0^. Finally, in the non-PEGylated system, FR is interacting with a higher number of FAs^b0^ and, due to the absence of the PEG spacer, is much more in contact with the NP, creating a kind of protein corona around it.

To assess the convergence of the simulations, in Figure S6, we report the time evolution of the TiO_2_-LIG^b0^ and TiO_2_–FR COM distances along the MD simulations for the three systems. We observe that convergence is reached in a shorter time for the non-PEGylated system due to the reduced mobility of the rigid FAs^b0^, covalently bonded to the NP, compared to when they are conjugated to PEG chains. We have also determined the extent of interaction between the functionalized NP and the FR by calculating their contact surface area along the MD simulation. The trends are shown in Figure S7. In particular, for the PEGylated systems, the contact surface area increases with time since the PEG chains tend to coil, and therefore, the FR can get closer to the NP. Moreover, the contact surface area is larger for FR/TiO_2_–PEG-20-FAs^b0^ than FR/TiO_2_–PEG-10-FAs^b0^: this can be explained by a more polar environment around the NP at higher FAs^b0^ density, which favors the interaction with the protein holding a net positive charge. On the contrary, in the case of the FR/TiO_2_-48-FAs^b0^-γ system, the contact surface area decreases along the simulation and, at the end, sets at a value that is twice that for the PEGylated systems. Hence, the effect of the PEG chains is that of decreasing the contact surface area between the protein and the functionalized nanodevice. Moreover, regarding the non-PEGylated system, it is reasonable that the protein optimizes its arrangement around the functionalized NP. This conformational rearrangement could potentially involve changes in the protein secondary structure.

In order to get further insight into this aspect, we performed a secondary structure analysis on FR along the 200 ns MD simulations for the FR/TiO_2_–PEG-10-FAs^b0^, FR/TiO_2_–PEG-20-FAs^b0^, and FR/TiO_2_-48-FAs^b0^-γ systems: the results are reported in Figure S8. In general, all six α-helices are conserved along the simulations, except for the one in the region comprised by residues 164–170, which alternates with H-bonded turns during the first 150 ns but is stable until the end of the simulation in the FR/TiO_2_–PEG-20-FAs^b0^ system. The four β-sheets are well conserved in the PEGylated systems, especially in the FR/TiO_2_–PEG-10-FAs^b0^ one. On the contrary, in the FR/TiO_2_-48-FAs^b0^-γ system, 2 β-sheets are lost at residues 92–98 and 104–110. Therefore, in the non-PEGylated system, we observe no perturbation of the α-helices but only the loss of 2 β-sheets upon interaction with the NP. This change in the protein conformation results from the intimate contact between FR and the functionalized NP when there are no PEG chains acting as molecular spacers.

**Figure 6:**
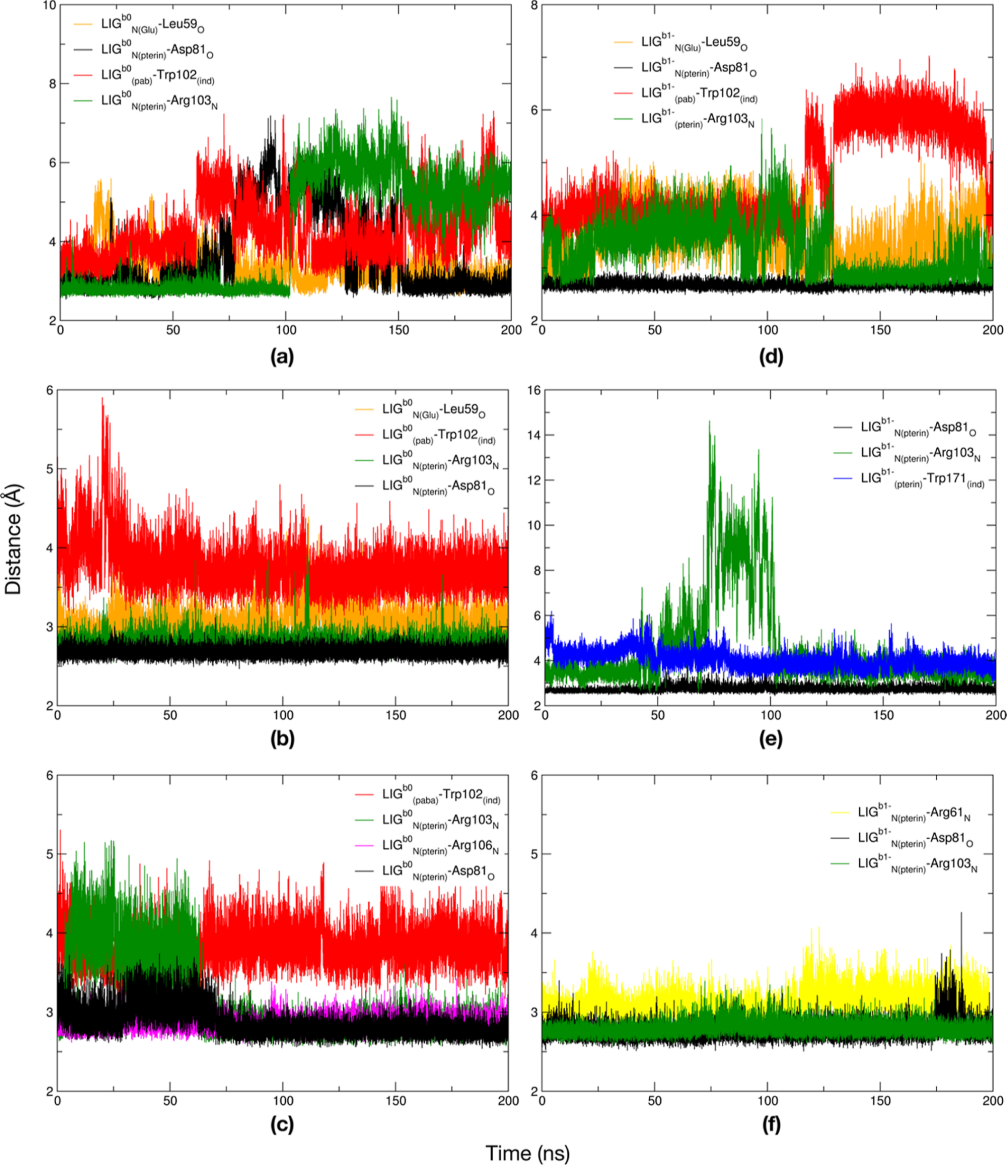
Time evolution of distances between selected atoms of LIG^b0,1–^ and FR AAs for the 200 ns simulations of the FR/TiO_2_–PEG-10-FAs^b0^ (a), FR/TiO_2_–PEG-20-FAs^b0^ (b), FR/TiO_2_-48-FAs^b0^-γ (c), FR/TiO_2_–PEG-10-FAs^b1–^ (d), FR/TiO_2_–PEG-20-FAs^b1–^ (e), and FR/TiO_2_-48-FAs^b1–^-γ (f) systems in physiological environment.

To focus on the dynamics of the active site, we conducted a similar analysis to that of Section 3.2, determining the ligand arrangement inside the binding pocket as a function of time, for the 200 ns MD simulations of the FR/TiO_2_–PEG-10-FAs^b0^, FR/TiO_2_–PEG-20-FAs^b0^, and FR/TiO_2_-48-FAs^b0^-γ systems (Figure 6a–c). In general, we observe a more sustained oscillation of distance values for FR/TiO_2_–PEG-10-FAs^b0^ (Figure 6a) rather than FR/TiO_2_–PEG-20-FAs^b0^ (Figure 6b) or FR/TiO_2_-48-FAs^b0^-γ (Figure 6c). Compared to the MD simulations without the NP (Section 3.2), we register a reduction in the number of AAs of the pocket interacting with the ligand, although the interaction with Asp81 (the fundamental one) and those with Trp102 and Arg103 both survive. Therefore, the presence of the NP perturbs the dynamics of the ligand in the binding site but only marginally, given that LIG^b0^ remains in the pocket all over the 200 ns long simulation for all the three systems.

In Table S3, instead, we analyze the last 50 ns dynamics of the FR/TiO_2_–PEG-10-FAs^b0^, FR/TiO_2_–PEG-20-FAs^b0^, and FR/TiO_2_-48-FAs^b0^-γ systems from the energetic point of view and in terms of the number of H-bonds formed. Regarding the LIG^b0^-FR interactions, while vdW and electrostatic energies are comparable in FR/TiO_2_–PEG-10-FAs^b0^, the contribution is majorly electrostatic in the FR/TiO_2_–PEG-20-FAs^b0^ and FR/TiO_2_-48-FAs^b0^-γ systems. One can notice that the LIG^b0^-FR interaction is maximized in the presence of the spacer and at the highest FA density, which is probably due to the fact that the binding pocket is not deformed due to excessive TiO_2_–FR contact, as it is in the case of the TiO_2_-48-FAs^b0^-γ system, and more FAs^b0^ decrease the LIG^b0^-FR distance, compared to the TiO_2_–PEG-10-FAs^b0^ system.

**Figure 7:**
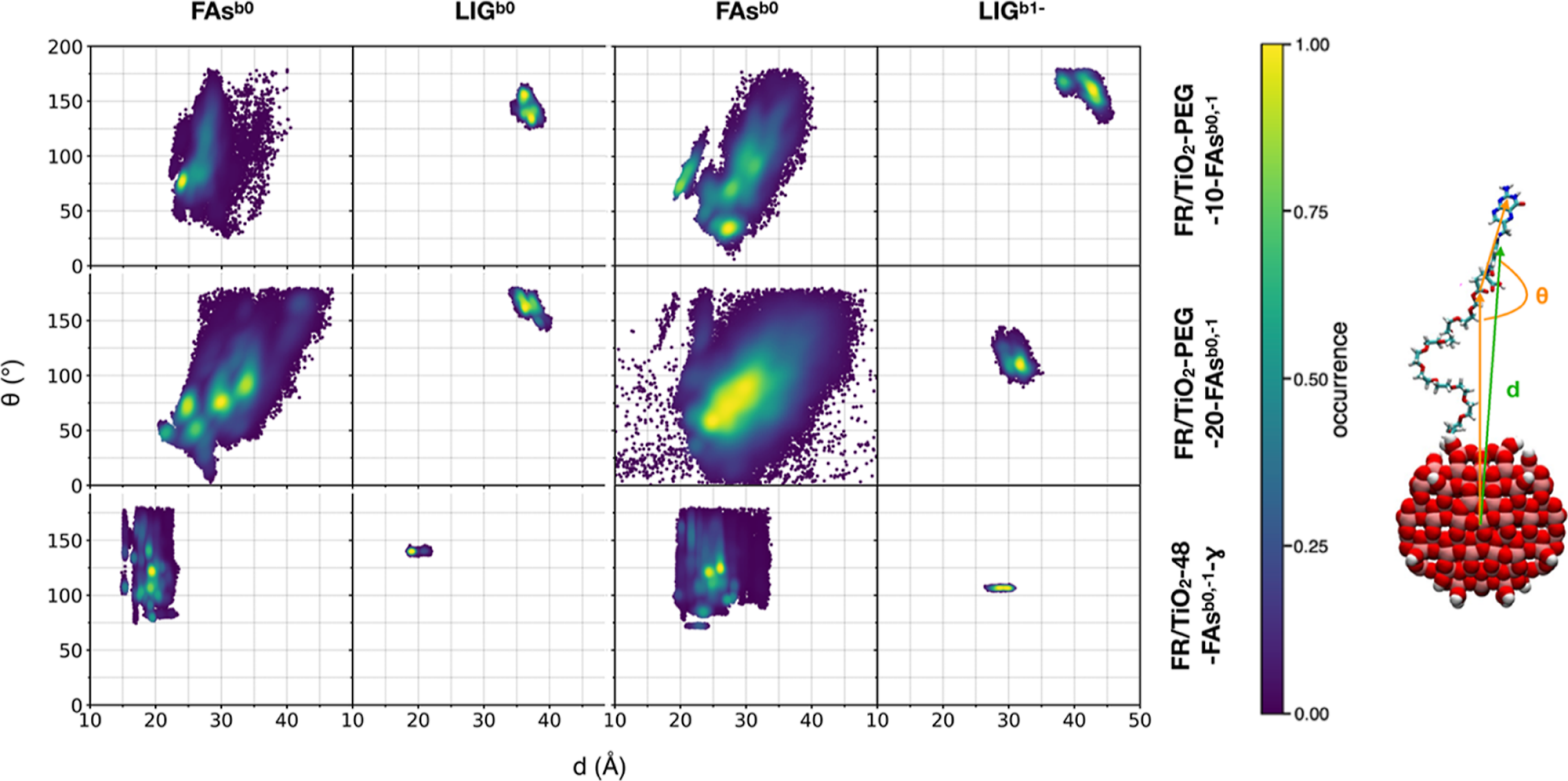
Conformational analysis on FA orientation with respect to the TiO_2_ NP along the last 50 ns of the 200 ns MD simulations of the FR/TiO_2_–PEG-10-FAs^b0^, FR/TiO_2_–PEG-20-FAs^b0^, FR/TiO_2_-48-FAs^b0^-γ, FR/TiO_2_–PEG-10-FAs^b1–^, FR/TiO_2_–PEG-20-FAs^b1–^, and FR/TiO_2_-48-FAs^b1–^-γ systems in physiological environment. As shown on the right, the distance *d* is between the Ti atom at the center of the NP and each FA^b^ (or LIG^b^) COM (green line), the angle *Θ* is the one formed by the NP central Ti atom, the C atom of each FAb (or LIGb) γ-carboxyl group, and the C atom of each FA*b* (or LIG*b*) in the pterin portion bringing the amino group, respectively (orange lines).

In Figure 7, we analyze how the FA molecules arrange with respect to the NP along the last 50 ns of the 200 ns MD simulations of the FR/TiO_2_–PEG-10-FAs^b0^, FR/TiO_2_–PEG-20-FAs^b0^, and FR/TiO_2_-48-FAs^b0^-γ systems. In particular, in a 2D graph, we plotted the angle θ formed by the NP central Ti atom, the C atom of each FA^b0^ (or LIG^b0^) γ-carboxyl group, and the C atom of each FA^b0^ (or LIG^b0^) pterin bringing the amino group (on the vertical axis) against the distance *d* of each FA COM from the Ti atom at the center of the NP (on the horizontal axis).

In the case of the PEGylated systems, we observe a greater number of recurrent conformations for the undocked FA molecules (FAs^b0^) at increasing FAs^b0^ density on the TiO_2_ NP. However, the opposite trend is observed for LIG^b0^. Indeed, for the system with 20 FAs^b0^, we found a single predominant arrangement of LIG^b0^ corresponding to a distance of 36–37 Å and an angle of 160–170°, whereas for that with 10 FAs^b0^, there is another frequently observed ligand conformation at a higher distance (37–38 Å) and a reduced angle (130–140°). These findings are in line with the results of Figure 6, where we found that the deviation of the distance values of LIG^b0^ inside the FR binding pocket is larger in the FR/TiO_2_–PEG-10-FAs^b0^ system, indicating that LIG^b0^ has a higher mobility, than in FR/TiO_2_–PEG-20-FAs^b0^. Finally, regarding the non-PEGylated system in Figure 7, we register a much lower conformational variability for the 47 undocked FAs^b0^, whereas LIG^b0^ is found to lie at a short distance (18–19 Å) with a tilting angle of 140–145°.

Still related to structural parameters, in Figure S9, we report the radial distribution function profiles of PEG, FAs^b0^, LIG^b0^, and FR, calculated with respect to the Ti atom at the center of the NP on the last 50 ns of the 200 ns MD simulations of the FR/TiO_2_–PEG-10-FAs^b0^ (Figure S9a) and FR/TiO_2_–PEG-20-FAs^b0^ (Figure S9b) systems. These results, together with those of Table S3, reveal a stronger interaction between the FR and the functionalized NP at the highest FAs^b0^ density.

#### 3.3.2 Effect of the FA Deprotonation

In this last section, we perform MD simulations of the FR/TiO_2_–PEG-*n*-FAs^b1–^ and FR/TiO_2_-48-FAs^b1–^-γ systems at 303 K in a 0.15 M NaCl water solution, where LIG^b1–^ and all of the FAs^b1–^ molecules have their α-COOH group deprotonated. In Figure S10, we report the time evolution of the TiO_2_-LIG^b1–^ and TiO_2_–FR distances. In the case of the PEGylated nanosystem with 10 FAs^b1–^, the TiO_2_-LIG^b1–^ and TiO_2_–FR distances, initially set to have stretched PEG chains, after the first 20 ns of the MD simulation drop to about 40 and 50 Å, respectively, and oscillate around those values until 180 ns; then they slightly increase to 42–43 and 52–53 Å, respectively, causing the loss of some FR/PEG interactions. In the case of the PEGylated system with 20 FAs^b1–^, instead, the corresponding distances fluctuate around 30 and 40 Å, respectively. For the non-PEGylated system, TiO_2_–FR and TiO_2_-LIG^b1–^ distances converge to 26 and 38 Å, respectively. Moreover, in Figure S11, we report the FR/TiO_2_+PEG + FAs^b1–^ contact surface area computed along the 200 ns MD simulations.

Comparing the results in Figures S10 and S11 with those in Figures S6 and S7, we observe that distances, both taken from COM or rdf profiles in Figure S12, do not always correlate with the contact surface area. In FR/TiO_2_-48-FAs^b^-γ, the TiO_2_-LIG^b^ and TiO_2_–FR distances increase for FAs^b1–^ and LIG^b1–^, although the contact surface area is almost unchanged with respect to neutral. For FR/TiO_2_–PEG-10-FAs^b^, although distances increase, the contact surface areas increase too. Finally, for the FR/TiO_2_–PEG-20-FAs^b^ system, a distance decrease corresponds to a contact surface area increase.

Regarding the dynamics of LIG^b1–^ inside the FR binding pocket, we see from Figure 6d–f that in all the systems, LIG^b1–^ has a very stable interaction with FR Asp81 and a quite stable one with Arg103 along the last 50 ns of the simulation. Moreover, in FR/TiO_2_–PEG-10-FAs^b1–^, LIG^b1–^ interacts with Leu59 and Arg103 in the second half of the simulation. In the FR/TiO_2_–PEG-20-FAs^b1–^ and FR/TiO_2_-48-FAs^b1–^-γ systems, there is an additional stable interaction with Trp171 and Arg61, respectively. Comparing these results with the ones of LIG^b0^ (Figure 6a–c), we find a general agreement due to the fact that LIG^b1–^ stays inside the FR binding pocket all along the 200 ns MD simulations, strongly interacting with Asp81, and, as in the case of LIG^b0^, the oscillation of the distance values describing FR-LIG^b1–^ interactions results more sustained in FR/TiO_2_–PEG-10-FAs^b1–^ than FR/TiO_2_–PEG-20-FAs^b1–^ or FR/TiO_2_-48-FAs^b1–^-γ. Then, the interactions with other AAs are relatively similar.

In Table S4, we report the corresponding energetic analysis as in Table S3 for the systems with FAs^b1–^ and LIG^b1–^, considering the last 50 ns of the MD simulations. The most relevant differences, going from values in Table S3 to those in Table S4, are that the extent of the FAs^b^–FAs^b^ interactions is reduced since negatively charged molecules are less prone to interact among themselves rather than when they are neutral because of electrostatic repulsion; the TiO_2_–FAs^b^ electrostatic interactions increased, as well as the FAs^b^ interactions with FR. The FR-LIG^b1–^ interaction energy is larger for the FR/TiO_2_–PEG-20-FAs^b1–^ system than the FR/TiO_2_-48-FAs^b1–^-γ system. One may notice a particularly high FR-LIG^b1–^ interaction for the FR/TiO_2_–PEG-10-FAs^b1–^ system. However, this is probably due to an artifact of the MD simulation where Lys19 is found to interact with LIG^b1–^ instead of taking part in the H-bond network responsible for the protein secondary structure. Considering the overall nonbonding interactions between the nanodevice (TiO_2_–PEG-*n*-FAs^b1–^) and the FR, at the bottom of Table S4, we can conclude that the one at higher FAs^b1–^ coverage density is more interacting than that at lower coverage density (−265 vs −173 kcal mol^–1^). In the case of FR/TiO_2_-48-FAs^b1–^-γ, the interaction would be even larger (−298 kcal mol^–1^), but we have already discussed in Section 3.3.1 that this is due to a large deformation of the protein secondary structure, which is expected to cause a loss in the protein function.

We also report in Figure 7 the angle vs distance 2D plots for the three systems with LIG^b1–^ and FAs^b1–^ similarly as the ones shown for LIG^b0^ and FAs^b0^. In the case of FR/TiO_2_–PEG-10-FAs^b1–^, there is one recurrent conformation of LIG^b1–^ associated with a distance of 42–43 Å from the NP center and an angle of 160–170° and one recurrent conformation of FAs^b1–^ associated with a distance of 27–28 Å and an angle of 30–40°. In the case of FR/TiO_2_–PEG-20-FAs^b1–^, we observe a narrower maximum probability spot for LIG^b1–^ (at a distance of 32 Å and an angle of 110–115°) but a broader distribution for the FAs^b1–^ molecules (maximum probability at distances of 24–27 Å and angles of 55–75°). At last, for the FR/TiO_2_-48-FAs^b1–^-γ system, we identified two highly recurrent spots on the density map for FAs^b1–^ (one at a distance of 24 Å and an angle of 120°–125° and the other at a distance of 25 Å and an angle of 25°) and only one for LIG^b1–^ (at a distance of 27–30 Å and an angle of 100–110°).

To complete the analysis of this section, we have performed a secondary structure calculation on FR, reported in Figure S13, to check whether the conformation of the protein is altered because of the change in the protonation state of the FA molecules functionalized on the TiO_2_ NP. Once again, we can state that no relevant changes have been found, within the simulation time framework, except that residues between 200 and 214 alternate between an α-helix and a right-handed π-helix for most of the simulation in the FR/TiO_2_–PEG/10-FAs^b1–^ system and only for a short time in the FR/TiO_2_–PEG-20-FAs^b1–^ system.

In order to make our considerations more robust, we have performed a first replica of 200 ns MD simulation of FR/TiO_2_–PEG-20-FAs^b1–^ starting from the same equilibrated structure we used for the original simulation but assigning different initial velocities to the atoms and a second replica of 50 ns MD simulation of FR/TiO_2_–PEG-20-FAs^b1–^ starting from the structure at 150 ns of the original simulation but assigning different initial velocities to the atoms. The analysis of these two replicas is provided in the Supporting Information in terms of the corresponding images and discussion (Figures S14–S18 for the first replica and Figures S19–S23 for the second replica). Indeed, we observe that the three simulations provide a consistent scenario, which strengthens our conclusions.

## 4 Conclusions

In this work, we investigated the dynamics of PEGylated or non-PEGylated, FA-functionalized TiO_2_ NPs interacting with the FR by classical MD simulations.

As a preliminary step, we have focused on the receptor/ligand complex in the absence of the NP. In particular, we have considered three case studies, where the two carboxyl groups of LIG are either both protonated (LIG^0^) or the α-COOH group is deprotonated (LIG^1–^) or both are deprotonated (LIG^2–^) since the special environment of the FR binding pocket could in principle allocate the ligand at different protonation states. Our docking and free energy calculations have shown a higher affinity of FR for the deprotonated forms of LIG than for the neutral one, specifically LIG^2–^ > LIG^1–^ > LIG^0^. We have also found a good accordance between our calculations and existing experimental^9^ and theoretical^14^ works in terms of binding free energies. These results suggest that the most probable protonation state of LIG when docked in the FR pocket at physiological pH is either −2 or −1. The free energy results are in agreement with the SOM analysis, where we found that the protonation state of the second carboxylic group of LIG (γ) has a lesser impact on the stability of the binding modes compared to that of the deprotonation of the first –COOH group (α).

Then, the main body of the work concerns a roundish TiO_2_ NP decorated with many FAs^b^ molecules, either by direct covalent binding to the surface (47 FAs^b^) or through some PEG polymer chains acting as molecular spacers (9 or 19 FAs^b^, depending on the coverage density). Our MD simulations have revealed that among the two PEGylated systems considered, the one with the highest FAs^b^ density has a greater interaction with the FR due to a more pronounced polar environment around the PEGylated NP. In the absence of the PEG spacers, we observe an excessive interaction between the nanodevice and the FR, leading to a larger protein deformation due to a protein corona effect.

We analyzed the effect of the protonation state of FA molecules on the NP interaction with FR by comparing FAs^b0^ and FAs^b1–^ (FAs^b2–^ cannot be considered because γ-COOH is used as an anchoring group). Compared to the case with FAs^b0^ and LIG^b0^, the two most interesting observations are that the contact surface area between the functionalized NP and the FR is boosted, especially for the PEGylated system with the highest FAs^b^ content, and that the FR–FAs^b^ interactions increase, whereas the FAs^b^–FAs^b^ ones decrease, which denote a better capability of the functionalized NP to target FR. The protein conformational analysis has also confirmed that the FR secondary structure is not significantly altered by the presence of the ligand in the pocket or even of the NP along the MD simulation, and the most evident changes have been registered with the non-PEGylated NP.

In conclusion, we envision that this pioneering computational work provides not only a better understanding of the molecular origin of ligand recognition to its receptor but also valuable information at an atomistic level of resolution on the effect of the presence of an attached inorganic NP, acting as a FA carrier, on the FA binding to the FR.

## Supporting Information Available

LIG binding energies to FR; FR secondary structure analysis; number of hydrogen bonds and nonbonded interaction energies; PMF profiles and umbrella histograms; set of atoms used for SOM training and Silhouette profile; TiO_2_-LIG and TiO_2_–FR COM distances; nanodevice–FR contact surface area; radial distribution functions; and replicas of MD simulation of the FR/TiO_2_–PEG-20-FAs^b1–^ system (PDF)

## References

[ref1] Sinha R.; Kim G. J.; Nie S.; Shin D. M. Nanotechnology in Cancer Therapeutics: Bioconjugated Nanoparticles for Drug Delivery. Mol. Cancer Ther. 2006, 5 (8), 1909–1917. 10.1158/1535-7163.MCT-06-0141. 16928810

[ref2] Leamon C. P.; Low P. S. Folate-Mediated Targeting: From Diagnostics to Drug and Gene Delivery. Drug Discovery Today 2001, 6 (1), 44–51. 10.1016/S1359-6446(00)01594-4. 11165172

[ref3] Lu Y.; Low P. S. Folate-Mediated Delivery of Macromolecular Anticancer Therapeutic Agents. Adv. Drug Delivery Rev. 2002, 54 (5), 675–693. 10.1016/S0169-409X(02)00042-X. 12204598

[ref4] Sudimack J.; Lee R. J. Targeted Drug Delivery via the Folate Receptor. Adv. Drug Delivery Rev. 2000, 41 (2), 147–162. 10.1016/S0169-409X(99)00062-9. 10699311

[ref5] Hilgenbrink A. R.; Low P. S. Folate Receptor-Mediated Drug Targeting: From Therapeutics to Diagnostics. J. Pharm. Sci. 2005, 94 (10), 2135–2146. 10.1002/jps.20457. 16136558

[ref6] Zwicke G. L.; Ali Mansoori G.; Jeffery C. J. Utilizing the Folate Receptor for Active Targeting of Cancer Nanotherapeutics. Nano Rev. 2012, 3 (1), 18496 10.3402/nano.v3i0.18496. PMC3521101 23240070

[ref7] Bandara N. A.; Hansen M. J.; Low P. S. Effect of Receptor Occupancy on Folate Receptor Internalization. Mol. Pharmaceutics 2014, 11 (3), 1007–1013. 10.1021/mp400659t. 24446917

[ref8] Fernández M.; Javaid F.; Chudasama V. Advances in Targeting the Folate Receptor in the Treatment/Imaging of Cancers. Chem. Sci. 2018, 9 (4), 790–810. 10.1039/C7SC04004K. 29675145 PMC5890329

[ref9] Chen C.; Ke J.; Zhou X. E.; Yi W.; Brunzelle J. S.; Li J.; Yong E.-L.; Xu H. E.; Melcher K. Structural Basis for Molecular Recognition of Folic Acid by Folate Receptors. Nature 2013, 500 (7463), 486–489. 10.1038/nature12327. 23851396 PMC5797940

[ref10] Wang C.; Jiang Y.; Fei X.; Gu Y. Design and interaction mechanism of ligand targeted with folate receptor α and β. J. Phys. Org. Chem. 2018, 31 (1), e3719 10.1002/poc.3719.

[ref11] Jiang Y.; Wang C.; Zhang M.; Fei X.; Gu Y. Type and size effect of functional groups on the novel antifolate target recognition folate receptors α and β: Docking, molecular dynamics and MM/PBSA study. J. Mol. Graphics Modell. 2020, 100, 107663 10.1016/j.jmgm.2020.107663. 32659629

[ref12] Wang C.; Jiang Y.; Zhang M.; Fei X.; Gu Y. Novel fluorescent antifolates that target folate receptors α and β: Molecular dynamics and density functional theory study. J. Mol. Graphics Modell. 2018, 85, 40–47. 10.1016/j.jmgm.2018.07.011. 30055477

[ref13] Jiang Y.; Wang C.; Zhang M.; Fei X.; Gu Y. Interacted Mechanism of Functional Groups in Ligand Targeted with Folate Receptor via Docking, Molecular Dynamic and MM/PBSA. J. Mol. Graphics Modell. 2019, 87, 121–128. 10.1016/j.jmgm.2018.12.003. 30537642

[ref14] Della-Longa S.; Arcovito A. Intermediate states in the binding process of folic acid to folate receptor α: insights by molecular dynamics and metadynamics. J. Comput.-Aided Mol. Des. 2015, 29 (1), 23–35. 10.1007/s10822-014-9801-8. 25323390

[ref15] Schaber E. N.; Ivanova N.; Iliev S.; Petrova J.; Gocheva G.; Madjarova G.; Ivanova A. Initial Stages of Spontaneous Binding of Folate-Based Vectors to Folate Receptor-α Observed by Unbiased Molecular Dynamics. J. Phys. Chem. B 2021, 125 (28), 7598–7612. 10.1021/acs.jpcb.1c00488. 34247488

[ref16] Gabizon A. Tumor Cell Targeting of Liposome-Entrapped Drugs with Phospholipid-Anchored Folic Acid-PEG Conjugates. Adv. Drug Delivery Rev. 2004, 56 (8), 1177–1192. 10.1016/j.addr.2004.01.011. 15094214

[ref17] Patri A.; Kukowskalatallo J.; Bakerjr J. Targeted Drug Delivery with Dendrimers: Comparison of the Release Kinetics of Covalently Conjugated Drug and Non-Covalent Drug Inclusion Complex. Adv. Drug Delivery Rev. 2005, 57 (15), 2203–2214. 10.1016/j.addr.2005.09.014. 16290254

[ref18] Wang H.; Zhao P.; Liang X.; Gong X.; Song T.; Niu R.; Chang J. Folate-PEG Coated Cationic Modified Chitosan - Cholesterol Liposomes for Tumor-Targeted Drug Delivery. Biomaterials 2010, 31 (14), 4129–4138. 10.1016/j.biomaterials.2010.01.089. 20163853

[ref19] Lan J.-S.; Liu L.; Zeng R.-F.; Qin Y.-H.; Hou J.-W.; Xie S.-S.; Yue S.; Yang J.; Ho R. J. Y.; Ding Y.; Zhang T. Tumor-Specific Carrier-Free Nanodrugs with GSH Depletion and Enhanced ROS Generation for Endogenous Synergistic Anti-Tumor by a Chemotherapy-Photodynamic Therapy. Chem. Eng. J. 2021, 407, 127212 10.1016/j.cej.2020.127212.

[ref20] Xu X.; Liu A.; Liu S.; Ma Y.; Zhang X.; Zhang M.; Zhao J.; Sun S.; Sun X. Application of Molecular Dynamics Simulation in Self-Assembled Cancer Nanomedicine. Biomater. Res. 2023, 27 (1), 39 10.1186/s40824-023-00386-7. 37143168 PMC10161522

[ref21] Porta F.; Lamers G. E. M.; Morrhayim J.; Chatzopoulou A.; Schaaf M.; den Dulk H.; Backendorf C.; Zink J. I.; Kros A. Folic Acid-Modified Mesoporous Silica Nanoparticles for Cellular and Nuclear Targeted Drug Delivery. Adv. Healthcare Mater. 2013, 2 (2), 281–286. 10.1002/adhm.201200176. 23184490

[ref22] Sun X.; Du R.; Zhang L.; Zhang G.; Zheng X.; Qian J.; Tian X.; Zhou J.; He J.; Wang Y.; Wu Y.; Zhong K.; Cai D.; Zou D.; Wu Z. A PH-Responsive Yolk-Like Nanoplatform for Tumor Targeted Dual-Mode Magnetic Resonance Imaging and Chemotherapy. ACS Nano 2017, 11 (7), 7049–7059. 10.1021/acsnano.7b02675. 28665575

[ref23] Lai T.-Y.; Lee W.-C. Killing of Cancer Cell Line by Photoexcitation of Folic Acid-Modified Titanium Dioxide Nanoparticles. J. Photochem. Photobiol., A 2009, 204 (2–3), 148–153. 10.1016/j.jphotochem.2009.03.009.

[ref24] Liang X.; Xie Y.; Wu J.; Wang J.; Petković M.; Stepić M.; Zhao J.; Ma J.; Mi L. Functional Titanium Dioxide Nanoparticle Conjugated with Phthalocyanine and Folic Acid as a Promising Photosensitizer for Targeted Photodynamic Therapy in Vitro and in Vivo. J. Photochem. Photobiol., B 2021, 215, 112122 10.1016/j.jphotobiol.2020.112122. 33433386

[ref25] Xie J.; Pan X.; Wang M.; Yao L.; Liang X.; Ma J.; Fei Y.; Wang P.-N.; Mi L. Targeting and Photodynamic Killing of Cancer Cell by Nitrogen-Doped Titanium Dioxide Coupled with Folic Acid. Nanomaterials 2016, 6 (6), 113 10.3390/nano6060113. 28335242 PMC5302625

[ref26] Ai J.; Liu B.; Liu W. Folic Acid-Tagged Titanium Dioxide Nanoparticles for Enhanced Anticancer Effect in Osteosarcoma Cells. Mater. Sci. Eng., C 2017, 76, 1181–1187. 10.1016/j.msec.2017.03.027. 28482484

[ref27] Naghibi S.; Madaah Hosseini H. R.; Faghihi Sani M. A.; Shokrgozar M. A.; Mehrjoo M. Mortality Response of Folate Receptor-Activated, PEG-Functionalized TiO_2_ Nanoparticles for Doxorubicin Loading with and without Ultraviolet Irradiation. Ceram. Int. 2014, 40 (4), 5481–5488. 10.1016/j.ceramint.2013.10.136.

[ref28] Shah Z.; Nazir S.; Mazhar K.; Abbasi R.; Samokhvalov I. M. PEGylated Doped- and Undoped-TiO_2_ Nanoparticles for Photodynamic Therapy of Cancers. Photodiagn. Photodyn. Ther. 2019, 27, 173–183. 10.1016/j.pdpdt.2019.05.019. 31136827

[ref29] Devanand Venkatasubbu G.; Ramasamy S.; Ramakrishnan V.; Kumar J. Folate Targeted PEGylated Titanium Dioxide Nanoparticles as a Nanocarrier for Targeted Paclitaxel Drug Delivery. Adv. Powder Technol. 2013, 24 (6), 947–954. 10.1016/j.apt.2013.01.008.

[ref30] Donadoni E.; Siani P.; Frigerio G.; Di Valentin C. Multi-Scale Modeling of Folic Acid-Functionalized TiO_2_ Nanoparticles for Active Targeting of Tumor Cells. Nanoscale 2022, 14 (33), 12099–12116. 10.1039/D2NR02603A. 35959762 PMC9404434

[ref31] Friesner R. A.; Murphy R. B.; Repasky M. P.; Frye L. L.; Greenwood J. R.; Halgren T. A.; Sanschagrin P. C.; Mainz D. T. Extra Precision Glide: Docking and Scoring Incorporating a Model of Hydrophobic Enclosure for Protein-Ligand Complexes. J. Med. Chem. 2006, 49 (21), 6177–6196. 10.1021/jm051256o. 17034125

[ref32] Madhavi Sastry G.; Adzhigirey M.; Day T.; Annabhimoju R.; Sherman W. Protein and Ligand Preparation: Parameters, Protocols, and Influence on Virtual Screening Enrichments. J. Comput.-Aided Mol. Des. 2013, 27 (3), 221–234. 10.1007/s10822-013-9644-8. 23579614

[ref33] Gocheva G.; Petkov N.; Garcia Luri A.; Iliev S.; Ivanova N.; Petrova J.; Mitrev Y.; Madjarova G.; Ivanova A. Tautomerism in Folic Acid: Combined Molecular Modelling and NMR Study. J. Mol. Liq. 2019, 292, 111392 10.1016/j.molliq.2019.111392.

[ref34] Halgren T. A.; Murphy R. B.; Friesner R. A.; Beard H. S.; Frye L. L.; Pollard W. T.; Banks J. L. Glide: A New Approach for Rapid, Accurate Docking and Scoring. 2. Enrichment Factors in Database Screening. J. Med. Chem. 2004, 47 (7), 1750–1759. 10.1021/jm030644s. 15027866

[ref35] Banks J. L.; Beard H. S.; Cao Y.; Cho A. E.; Damm W.; Farid R.; Felts A. K.; Halgren T. A.; Mainz D. T.; Maple J. R.; Murphy R.; Philipp D. M.; Repasky M. P.; Zhang L. Y.; Berne B. J.; Friesner R. A.; Gallicchio E.; Levy R. M. Integrated Modeling Program, Applied Chemical Theory (IMPACT). J. Comput. Chem. 2005, 26 (16), 1752–1780. 10.1002/jcc.20292. 16211539 PMC2742605

[ref36] Onufriev A. V.; Case D. A. Generalized Born Implicit Solvent Models for Biomolecules. Annu. Rev. Biophys. 2019, 48 (1), 275–296. 10.1146/annurev-biophys-052118-115325. 30857399 PMC6645684

[ref37] Phillips J. C.; Hardy D. J.; Maia J. D. C.; Stone J. E.; Ribeiro J. v.; Bernardi R. C.; Buch R.; Fiorin G.; Hénin J.; Jiang W.; McGreevy R.; Melo M. C. R.; Radak B. K.; Skeel R. D.; Singharoy A.; Wang Y.; Roux B.; Aksimentiev A.; Luthey-Schulten Z.; Kalé L. V.; Schulten K.; Chipot C.; Tajkhorshid E. Scalable Molecular Dynamics on CPU and GPU Architectures with NAMD. J. Chem. Phys. 2020, 153 (4), 044130 10.1063/5.0014475. 32752662 PMC7395834

[ref38] Huang J.; MacKerell A. D. CHARMM36 All-Atom Additive Protein Force Field: Validation Based on Comparison to NMR Data. J. Comput. Chem. 2013, 34 (25), 2135–2145. 10.1002/jcc.23354. 23832629 PMC3800559

[ref39] Vanommeslaeghe K.; Raman E. P.; MacKerell A. D. Automation of the CHARMM General Force Field (CGenFF) II: Assignment of Bonded Parameters and Partial Atomic Charges. J. Chem. Inf. Model. 2012, 52 (12), 3155–3168. 10.1021/ci3003649. 23145473 PMC3528813

[ref40] Jorgensen W. L.; Chandrasekhar J.; Madura J. D.; Impey R. W.; Klein M. L. Comparison of Simple Potential Functions for Simulating Liquid Water. J. Chem. Phys. 1983, 79 (2), 926–935. 10.1063/1.445869.

[ref41] Jo S.; Kim T.; Iyer V. G.; Im W. CHARMM-GUI: A Web-Based Graphical User Interface for CHARMM. J. Comput. Chem. 2008, 29 (11), 1859–1865. 10.1002/jcc.20945. 18351591

[ref42] Tuckerman M.; Berne B. J.; Martyna G. J. Reversible Multiple Time Scale Molecular Dynamics. J. Chem. Phys. 1992, 97 (3), 1990–2001. 10.1063/1.463137.

[ref43] Darden T.; York D.; Pedersen L. Particle Mesh Ewald: An N ·log(N) Method for Ewald Sums in Large Systems. J. Chem. Phys. 1993, 98 (12), 10089–10092. 10.1063/1.464397.

[ref44] Ryckaert J.-P.; Ciccotti G.; Berendsen H. J. C. Numerical Integration of the Cartesian Equations of Motion of a System with Constraints: Molecular Dynamics of n-Alkanes. J. Comput. Phys. 1977, 23 (3), 327–341. 10.1016/0021-9991(77)90098-5.

[ref45] Andersen H. C. Rattle: A “Velocity” Version of the Shake Algorithm for Molecular Dynamics Calculations. J. Comput. Phys. 1983, 52 (1), 24–34. 10.1016/0021-9991(83)90014-1.

[ref46] Miyamoto S.; Kollman P. A. Settle: An Analytical Version of the SHAKE and RATTLE Algorithm for Rigid Water Models. J. Comput. Chem. 1992, 13 (8), 952–962. 10.1002/jcc.540130805.

[ref47] Martyna G. J.; Tobias D. J.; Klein M. L. Constant Pressure Molecular Dynamics Algorithms. J. Chem. Phys. 1994, 101 (5), 4177–4189. 10.1063/1.467468.

[ref48] Feller S. E.; Zhang Y.; Pastor R. W.; Brooks B. R. Constant Pressure Molecular Dynamics Simulation: The Langevin Piston Method. J. Chem. Phys. 1995, 103 (11), 4613–4621. 10.1063/1.470648.

[ref49] Plimpton S. Fast Parallel Algorithms for Short-Range Molecular Dynamics. J. Comput. Phys. 1995, 117 (1), 1–19. 10.1006/jcph.1995.1039.

[ref50] Fazio G.; Ferrighi L.; Di Valentin C. Spherical versus Faceted Anatase TiO_2_ Nanoparticles: A Model Study of Structural and Electronic Properties. J. Phys. Chem. C 2015, 119 (35), 20735–20746. 10.1021/acs.jpcc.5b06384.

[ref51] Selli D.; Fazio G.; Di Valentin C. Modelling Realistic TiO_2_ Nanospheres: A Benchmark Study of SCC-DFTB against Hybrid DFT. J. Chem. Phys. 2017, 147 (16), 164701 10.1063/1.4994165. 29096504

[ref52] Selli D.; Tawfilas M.; Mauri M.; Simonutti R.; Di Valentin C. Optimizing PEGylation of TiO_2_ Nanocrystals through a Combined Experimental and Computational Study. Chem. Mater. 2019, 31 (18), 7531–7546. 10.1021/acs.chemmater.9b02329. 31875864 PMC6924593

[ref53] Selli D.; Motta S.; Di Valentin C. Impact of Surface Curvature, Grafting Density and Solvent Type on the PEGylation of Titanium Dioxide Nanoparticles. J. Colloid Interface Sci. 2019, 555, 519–531. 10.1016/j.jcis.2019.07.106. 31404836

[ref54] Brandt E. G.; Lyubartsev A. P. Systematic Optimization of a Force Field for Classical Simulations of TiO_2_ -Water Interfaces. J. Phys. Chem. C 2015, 119 (32), 18110–18125. 10.1021/acs.jpcc.5b02669.

[ref55] Siani P.; Motta S.; Ferraro L.; Dohn A. O.; Di Valentin C. Dopamine-Decorated TiO_2_ Nanoparticles in Water: A QM/MM vs an MM Description. J. Chem. Theory Comput. 2020, 16 (10), 6560–6574. 10.1021/acs.jctc.0c00483. 32880452 PMC7735700

[ref56] Siani P.; Frigerio G.; Donadoni E.; Di Valentin C. Molecular Dynamics Simulations of cRGD-Conjugated PEGylated TiO_2_ Nanoparticles for Targeted Photodynamic Therapy. J. Colloid Interface Sci. 2022, 627, 126–141. 10.1016/j.jcis.2022.07.045. 35842963

[ref57] Jewett A. I.; Stelter D.; Lambert J.; Saladi S. M.; Roscioni O. M.; Ricci M.; Autin L.; Maritan M.; Bashusqeh S. M.; Keyes T.; Dame R. T.; Shea J.-E.; Jensen G. J.; Goodsell D. S. Moltemplate: A Tool for Coarse-Grained Modeling of Complex Biological Matter and Soft Condensed Matter Physics. J. Mol. Biol. 2021, 433 (11), 166841 10.1016/j.jmb.2021.166841. 33539886 PMC8119336

[ref58] Martínez L.; Andrade R.; Birgin E. G.; Martínez J. M. PACKMOL: A Package for Building Initial Configurations for Molecular Dynamics Simulations. J. Comput. Chem. 2009, 30 (13), 2157–2164. 10.1002/jcc.21224. 19229944

[ref59] Kamberaj H.; Low R. J.; Neal M. P. Time Reversible and Symplectic Integrators for Molecular Dynamics Simulations of Rigid Molecules. J. Chem. Phys. 2005, 122 (22), 224114 10.1063/1.1906216. 15974658

[ref60] Siani P.; Di Valentin C. Effect of Dopamine-Functionalization, Charge and PH on Protein Corona Formation around TiO 2 Nanoparticles. Nanoscale 2022, 14 (13), 5121–5137. 10.1039/D1NR07647G. 35302136 PMC8969454

[ref61] Humphrey W.; Dalke A.; Schulten K. VMD: Visual Molecular Dynamics. J. Mol. Graphics 1996, 14 (1), 33–38. 10.1016/0263-7855(96)00018-5. 8744570

[ref62] Rycroft C. H. VORO++: A three-dimensional Voronoi cell library in C++. Chaos 2009, 19 (4), 041111 10.1063/1.3215722. 20059195

[ref63] Roe D. R.; Cheatham T. E. PTRAJ and CPPTRAJ: Software for Processing and Analysis of Molecular Dynamics Trajectory Data. J. Chem. Theory Comput. 2013, 9 (7), 3084–3095. 10.1021/ct400341p. 26583988

[ref64] Romo T. D.; Leioatts N.; Grossfield A. Lightweight Object Oriented Structure Analysis: Tools for Building Tools to Analyze Molecular Dynamics Simulations. J. Comput. Chem. 2014, 35 (32), 2305–2318. 10.1002/jcc.23753. 25327784 PMC4227929

[ref65] Abraham M. J.; Murtola T.; Schulz R.; Páll S.; Smith J. C.; Hess B.; Lindahl E. GROMACS: High Performance Molecular Simulations through Multi-Level Parallelism from Laptops to Supercomputers. SoftwareX 2015, 1–2, 19–25. 10.1016/j.softx.2015.06.001.

[ref66] Roux B. The Calculation of the Potential of Mean Force Using Computer Simulations. Comput. Phys. Commun. 1995, 91 (1–3), 275–282. 10.1016/0010-4655(95)00053-I.

[ref67] Jakubec D.; Vondrášek J. Can All-Atom Molecular Dynamics Simulations Quantitatively Describe Homeodomain-DNA Binding Equilibria?. J. Chem. Theory Comput. 2019, 15 (4), 2635–2648. 10.1021/acs.jctc.8b01144. 30807142

[ref68] Kohonen T. Essentials of the Self-Organizing Map. Neural Networks 2013, 37, 52–65. 10.1016/j.neunet.2012.09.018. 23067803

[ref69] Bertho G.; Mantsyzov; Bouvier; Evrard-Todeschi Contact-Based Ligand-Clustering Approach for the Identification of Active Compounds in Virtual Screening. Adv. Appl. Bioinf. Chem. 2012, 5, 61 10.2147/AABC.S30881. PMC3459543 23055752

[ref70] Yang Y.; Yao K.; Repasky M. P.; Leswing K.; Abel R.; Shoichet B. K.; Jerome S. V. Efficient Exploration of Chemical Space with Docking and Deep Learning. J. Chem. Theory Comput. 2021, 17 (11), 7106–7119. 10.1021/acs.jctc.1c00810. 34592101

[ref71] Li T.; Motta S.; Stevens A. O.; Song S.; Hendrix E.; Pandini A.; He Y. Recognizing the Binding Pattern and Dissociation Pathways of the P300 Taz2-P53 TAD2 Complex. JACS Au 2022, 2 (8), 1935–1945. 10.1021/jacsau.2c00358. 36032526 PMC9400049

[ref72] Hendrix E.; Motta S.; Gahl R. F.; He Y. Insight into the Initial Stages of the Folding Process in Onconase Revealed by UNRES. J. Phys. Chem. B 2022, 126 (40), 7934–7942. 10.1021/acs.jpcb.2c04770. 36179061

[ref73] Motta S.; Pandini A.; Fornili A.; Bonati L. Reconstruction of ARNT PAS-B Unfolding Pathways by Steered Molecular Dynamics and Artificial Neural Networks. J. Chem. Theory Comput. 2021, 17 (4), 2080–2089. 10.1021/acs.jctc.0c01308. 33780250 PMC8047803

[ref74] Motta S.; Callea L.; Bonati L.; Pandini A. PathDetect-SOM: A Neural Network Approach for the Identification of Pathways in Ligand Binding Simulations. J. Chem. Theory Comput. 2022, 18 (3), 1957–1968. 10.1021/acs.jctc.1c01163. 35213804 PMC8908765

[ref75] Wu Z.; Li X.; Hou C.; Qian Y. Solubility of Folic Acid in Water at pH Values between 0 and 7 at Temperatures (298.15, 303.15, and 313.15) K. J. Chem. Eng. Data 2010, 55 (9), 3958–3961. 10.1021/je1000268.

