# Supplementary material for: Molecular Dynamics for the Optimal Design of Functionalized Nanodevices to Target Folate Receptors on Tumor Cells
Source: ACS Biomater Sci Eng. 2023 Oct 13;9(11):6123–37. doi: 10.1021/acsbiomaterials.3c00942 (PMC10646887; doi:10.1021/acsbiomaterials.3c00942)
Supplement: Supplementary file 1 — ab3c00942_si_001.pdf [file ab3c00942_si_001.pdf]

Supporting Information

Molecular dynamics for the optimal design of functionalized nanodevices to target folate receptors on tumor cells

Edoardo Donadoni,[a] Giulia Frigerio,[a] Paulo Siani,[a] Stefano Motta,[b] Jacopo Vertemara,[c] Luca De Gioia,[c] Laura Bonati,[b] Cristiana Di Valentin[a,d*]

[a] *Dipartimento di Scienza dei Materiali, Università di Milano-Bicocca, via R. Cozzi 55, 20125 Milano, Italy.*
[b] *Dipartimento di Scienze dell'Ambiente e del Territorio, Università di Milano-Bicocca, Piazza della Scienza 1, 20126 Milano, Italy.*
[c] *Dipartimento di Biotecnologie e Bioscienze, Università di Milano-Bicocca, Piazza della Scienza 1, 20126 Milano, Italy.*
[d] *BioNanoMedicine Center NANOMIB, Università di Milano-Bicocca, via R. Follereau 3, 20854 Vedano al Lambro, Italy.*
[*]*Corresponding author: cristiana.divalentin@unimib.it*

Table S1. Breakdown of LIG binding energies in the FR binding pocket from docking calculations in GBIS implicit water solvent for the FR/LIG$^{0,1-,2-}$ systems.

	**$\Delta E_{binding}$ (kcal mol^{-1})**		
	**FR/LIG^{0}**	**FR/LIG^{1-}**	**FR/LIG^{2-}**
Total	-34.418	-49.369	-56.586
Stretching	0.458	1.538	2.583
Bending	5.363	-2.489	-7.133
Torsional	6.532	4.928	7.866
Improper	1.088	0.789	1.095
vdW	-46.973	-32.944	-29.289
Electrostatic	-69.938	-280.772	-430.734
Solvation	69.052	259.582	399.025

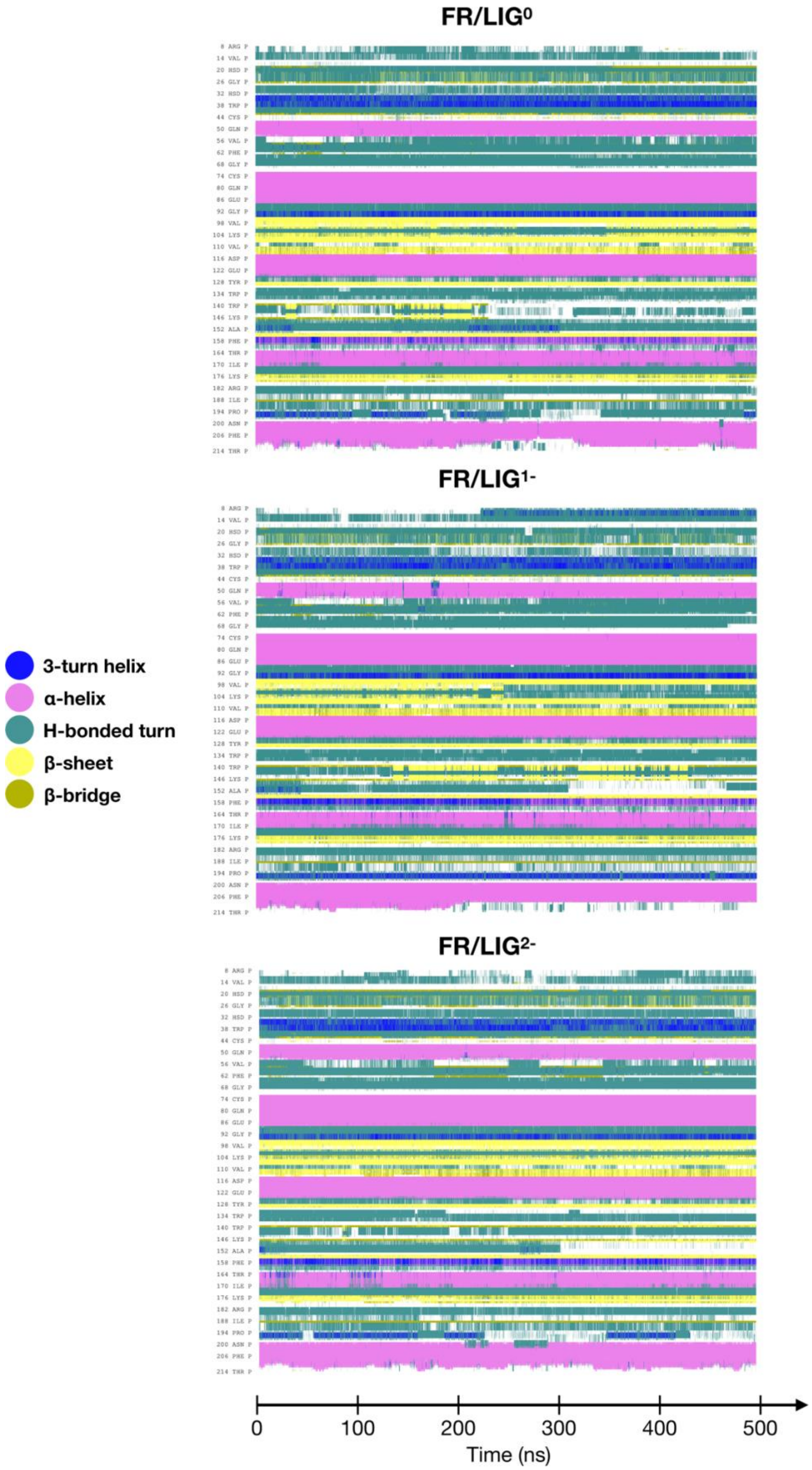

Figure S1. Secondary structure analysis performed along the 500 ns MD simulations of the FR/LIG$^{0,1-,2-}$ systems.

Table S2. Number of intermolecular hydrogen bonds and non-bonded interaction energies along the last 100 ns of the 500 ns MD production simulations of the FR/$LIG^{0,1-,2-}$ systems.

	**H-bonds number**	**Interaction energy (kcal mol^{-1})**	
		**VdW**	**Electrostatic**
	**FR/LIG^{0}**		
LIG^{0}-FR	0.4 (± 0.6)	-30 (± 21)	-58 (± 42)
LIG^{0}-wat	3 (± 2)	-4 (± 4)	-43 (± 17)
FR-wat	148 (± 32)	-307 (± 80)	-4849 (± 1042)
LIG^{0}-Leu59	0.04 (± 0.2)	-0.8 (± 1)	-2 (± 3)
LIG^{0}-Asp81	0.04 (± 0.2)	-0.7 (± 0.8)	-33 (± 5)
LIG^{0}-Tyr85	0.002 (± 0.04)	-3.8 (± 0.9)	3 (± 1)
LIG^{0}-Trp102	0.005 (± 0.07)	-7 (± 2)	-3 (± 2)
LIG^{0}-Arg103	0.3 (± 0.5)	-1 (± 2)	-34 (± 8)
LIG^{0}-Trp171	-	-7 (± 1)	3 (± 1)
LIG^{0}-Ser174	0.04 (± 0.2)	-0.6 (± 0.5)	0.3 (± 2)
	**FR/LIG^{1-}**		
LIG^{1-}-FR	1 (± 1)	-35 (± 9)	-174 (± 56)
LIG^{1-}-wat	5 (± 2)	-2 (± 5)	-118 (± 46)
FR-wat	174 (± 30)	-386 (± 73)	-5714 (± 978)
LIG^{1-}-Asp81	0.3 (± 0.5)	0.8 (± 2)	-34 (± 5)
LIG^{1-}-Tyr82	0.004 (± 0.07)	-1.1 (± 0.4)	-0.2 (± 2)
LIG^{1-}-Trp102	0.1 (± 0.2)	-3 (± 1)	-7 (± 5)
LIG^{1-}-Arg103	0.1 (± 0.3)	-1.1 (± 0.9)	-19 (± 12)
LIG^{1-}-His135	0.1 (± 0.3)	-3.0 (± 0.8)	4 (± 4)
LIG^{1-}-Lys136	0.2 (± 0.4)	-2 (± 2)	-70 (± 41)
LIG^{1-}-Gly137	0.4 (± 0.5)	-0.3 (± 1)	-15 (± 5)
LIG^{1-}-Trp171	-	-6.8 (± 0.9)	3 (± 1)
	**FR/LIG^{2-}**		
LIG^{2-}-FR	1 (± 1)	-28 (± 14)	-195 (± 102)
LIG^{2-}-wat	8 (± 2)	6 (± 5)	-280 (± 67)
FR-wat	154 (± 33)	-337 (± 80)	-5056 (± 1085)
LIG^{2-}-Asp81	0.2 (± 0.4)	0.1 (± 1)	-29 (± 9)
LIG^{2-}-Trp102	0.008 (± 0.09)	-2 (± 1)	-4 (± 4)
LIG^{2-}-Arg103	0.3 (± 0.5)	-0.3 (± 1)	-30 (± 19)
LIG^{2-}-Arg106	0.03 (± 0.2)	-0.3 (± 0.7)	-12 (± 8)
LIG^{2-}-Lys136	0.4 (± 0.5)	-0.3 (± 2)	-105 (± 37)
LIG^{2-}-Gly137	0.2 (± 0.4)	-0.3 (± 0.7)	-11 (± 7)
LIG^{2-}-Trp138	0.02 (± 0.1)	-2.0 (± 0.9)	-1 (± 4)
LIG^{2-}-Ser174	0.03 (± 0.2)	-0.4 (± 0.4)	-0.8 (± 3)

Specifically on the FR-LIG interactions in the receptor's binding pocket, we register a strong electrostatic interaction of LIG^{0} with the Asp81 and Arg103 residues, of LIG^{1-} with Asp81, Arg103, Lys136 and Gly137 and of LIG^{2-} with Asp81, Arg103, Arg106, Lys136 and Gly137. On the contrary, Van der Waals interactions are higher in magnitude for the neutral ligand and, in particular, with Tyr85, Trp102 and Trp171 residues.

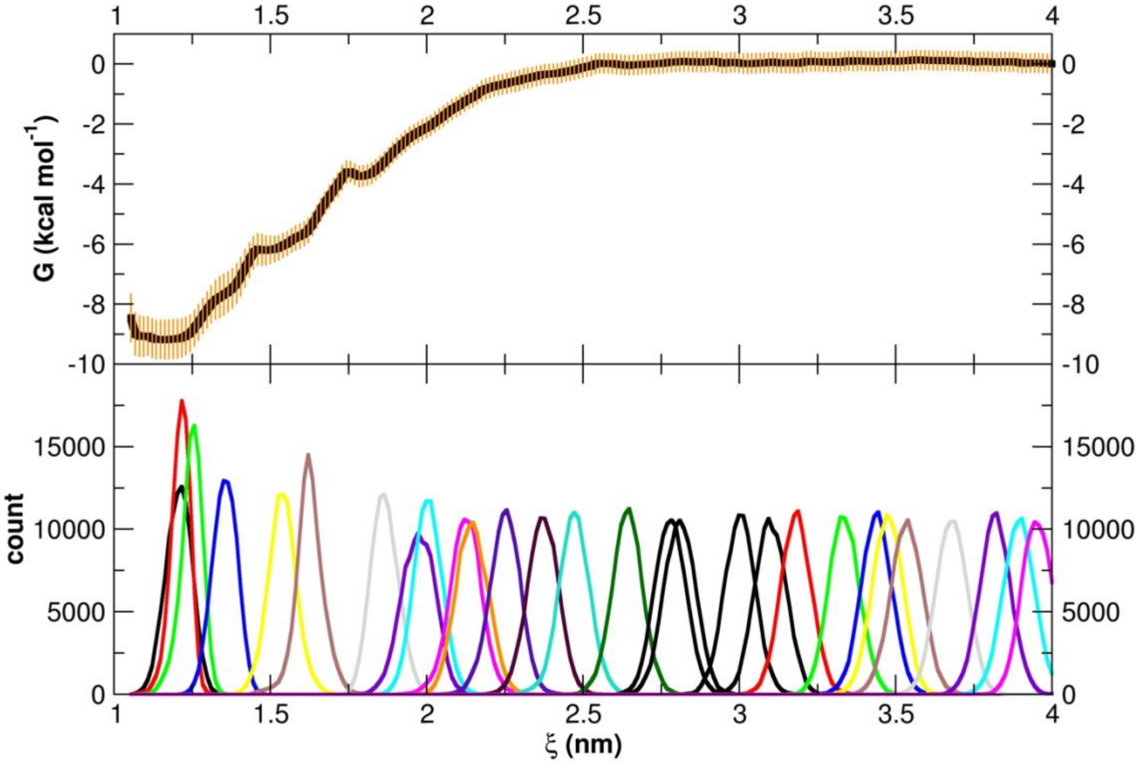

Figure S2. Top: PMF profile relative to LIG binding to FR for the FR/LIG^0 system (black line) with the associated error bar (orange). Bottom: umbrella histograms.

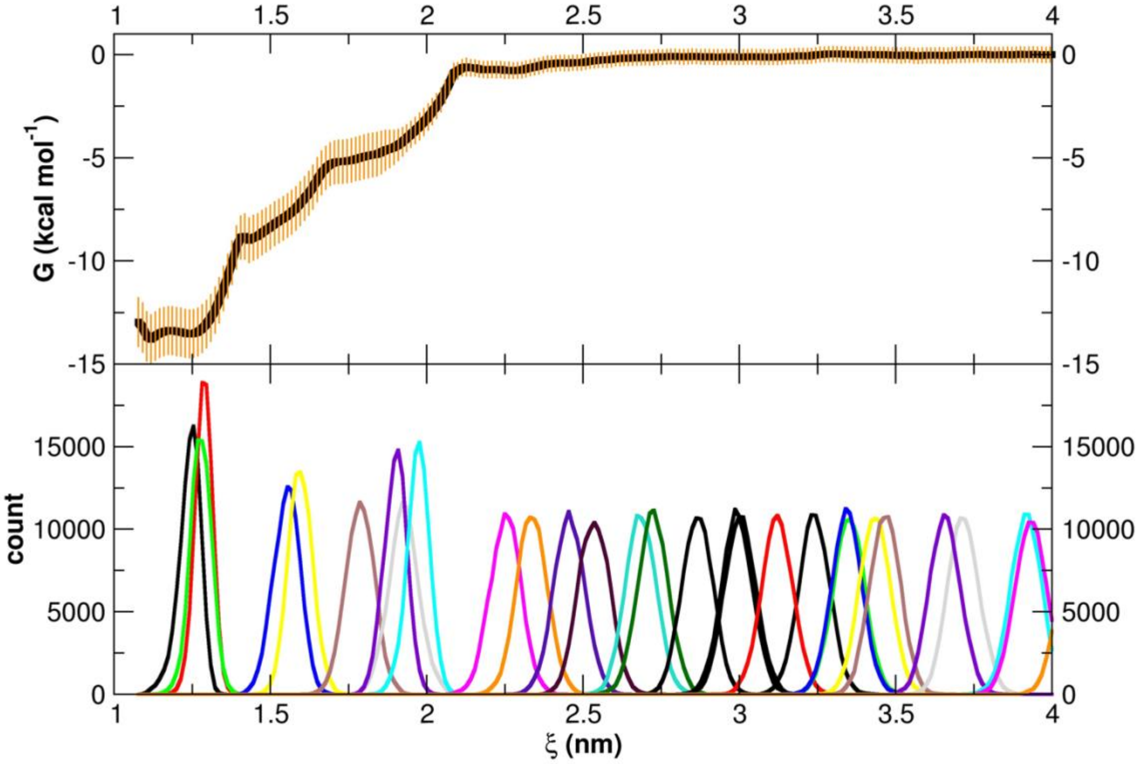

Figure S3. Top: PMF profile relative to LIG binding to FR for the FR/LIG^{1-} system (black line) with the associated error bar (orange). Bottom: umbrella histograms.

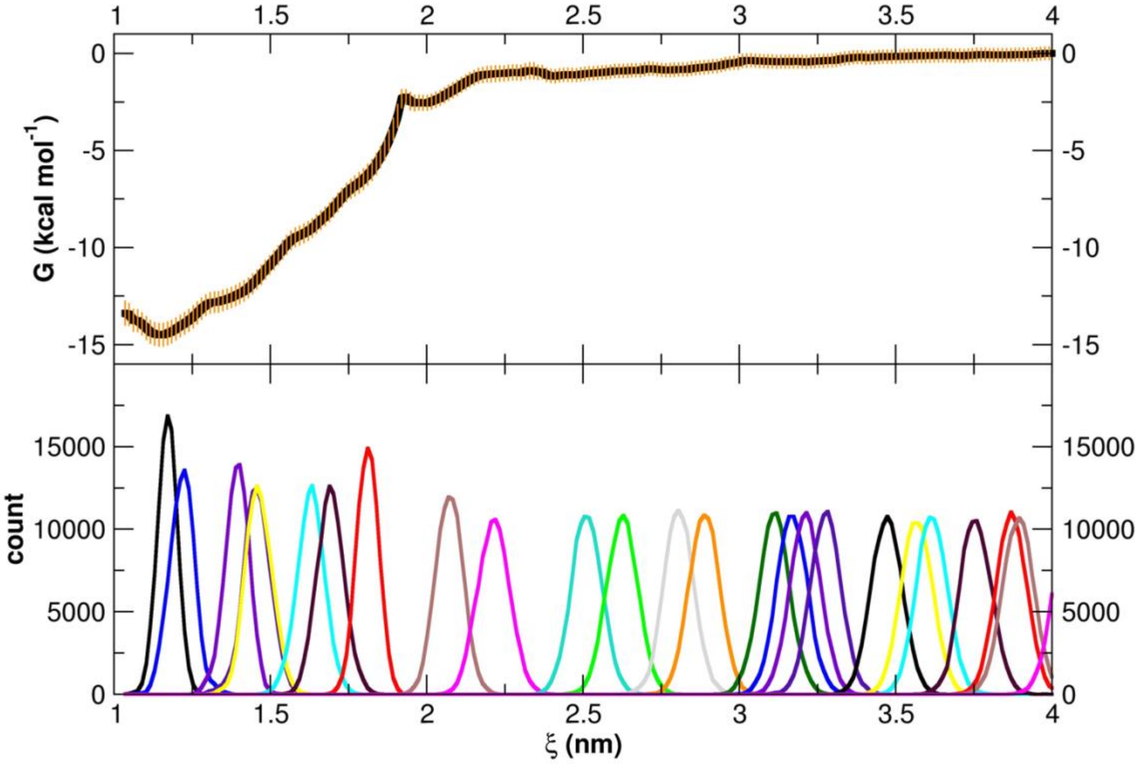

Figure S4. Top: PMF profile relative to LIG binding to FR for the FR/LIG^{2-} system (black line) with the associated error bar (orange). Bottom: umbrella histograms.

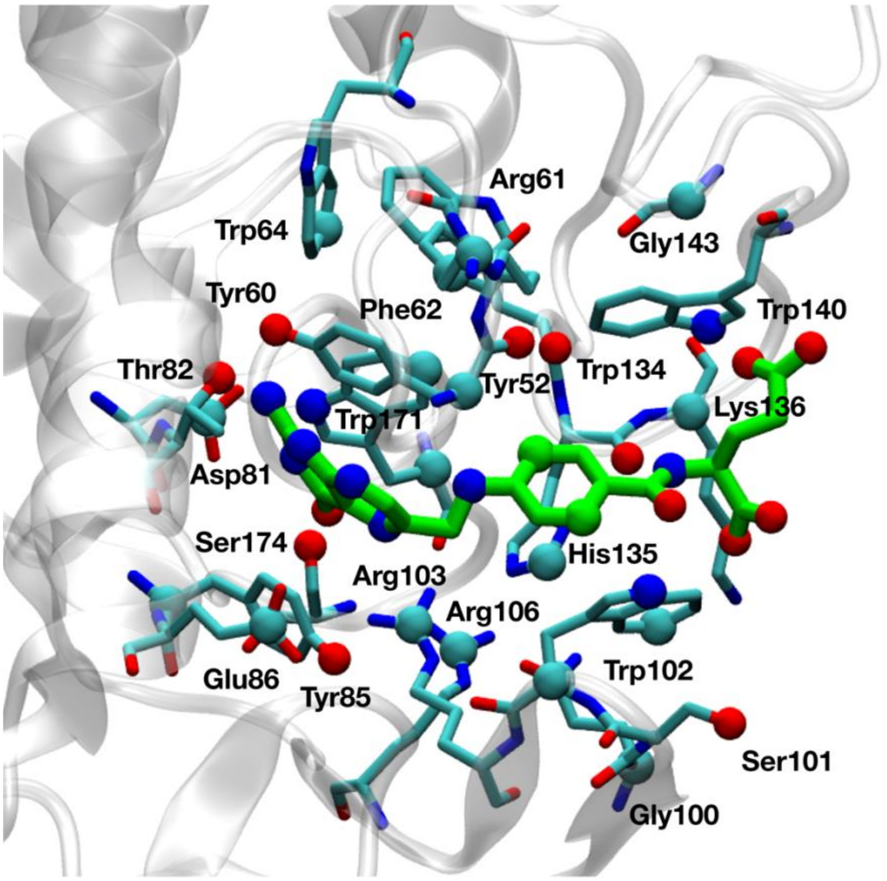

(a)

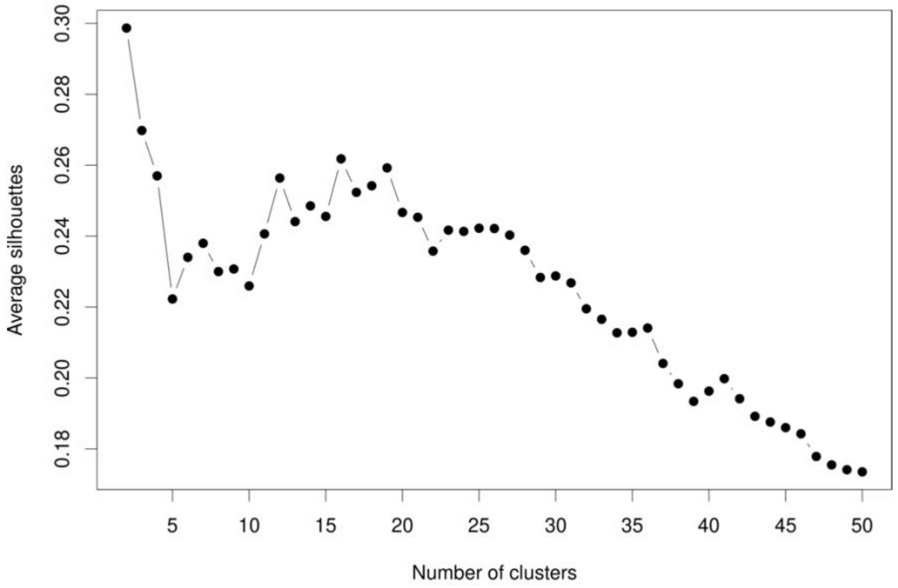

(b)

Figure S5. (a) Set of ligand and protein atoms (vdW representation) used to calculate intermolecular distances for SOM training. (b) Silhouette profile. The optimal number of clusters (7) was chosen as the one with the highest silhouette score in the range 5-10.

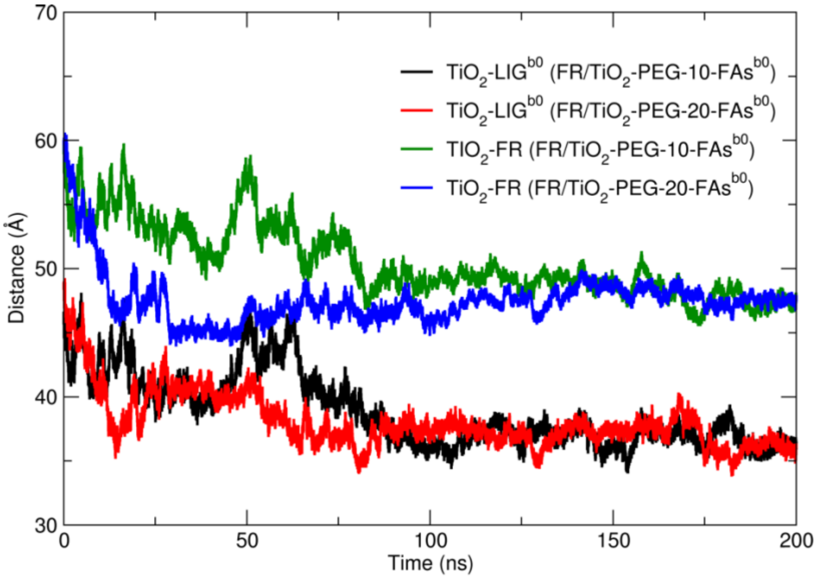

(a)

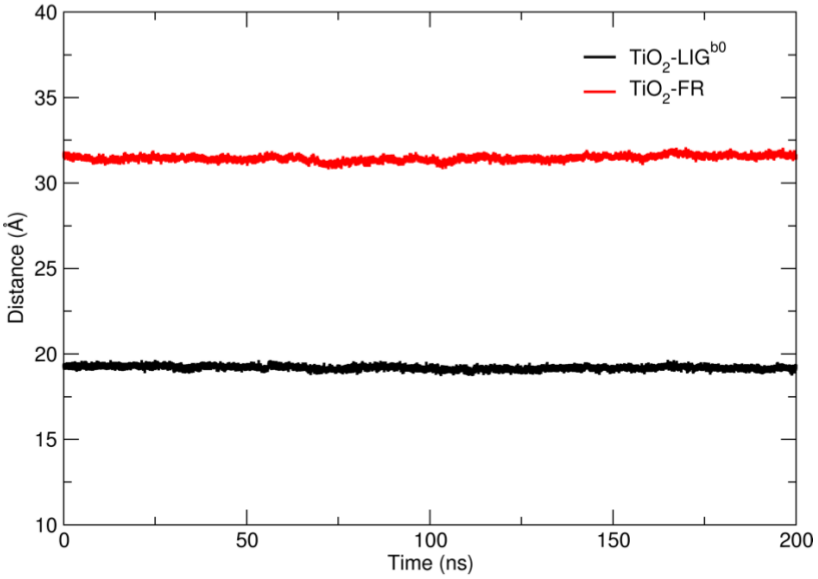

(b)

Figure S6. Time evolution of TiO_2-LIG^{b0} and TiO_2-FR centers of mass distances along the 200 ns MD simulations for the FR/TiO_2-PEG-10-FAs^{b0} and the FR/TiO_2-PEG-20-FAs^{b0} systems (a) and for the FR/TiO_2-48-FAs^{b0}-γ system (b).

We observe that for the PEGylated systems (**Figure S6a**), the PEG chains bend and the NP approaches the protein. Therefore, both TiO_2-LIG^{b0} and TiO_2-FR distances decrease until they stabilize after about 100 ns from the beginning of the simulation. In the case of the FR/TiO_2-48-FAs^{b0}-γ system (**Figure S6b**), the two distances are very much constant along the MD run, because LIG^{b0}, as well as all the other FAs^{b0}, is covalently bonded to the NP, and since it is a short rigid molecule, it has reduced mobility, therefore its COM is found in a narrow range of distances from the

NP center. Hence, it is trivial to understand that convergence is reached in a shorter time for this system.

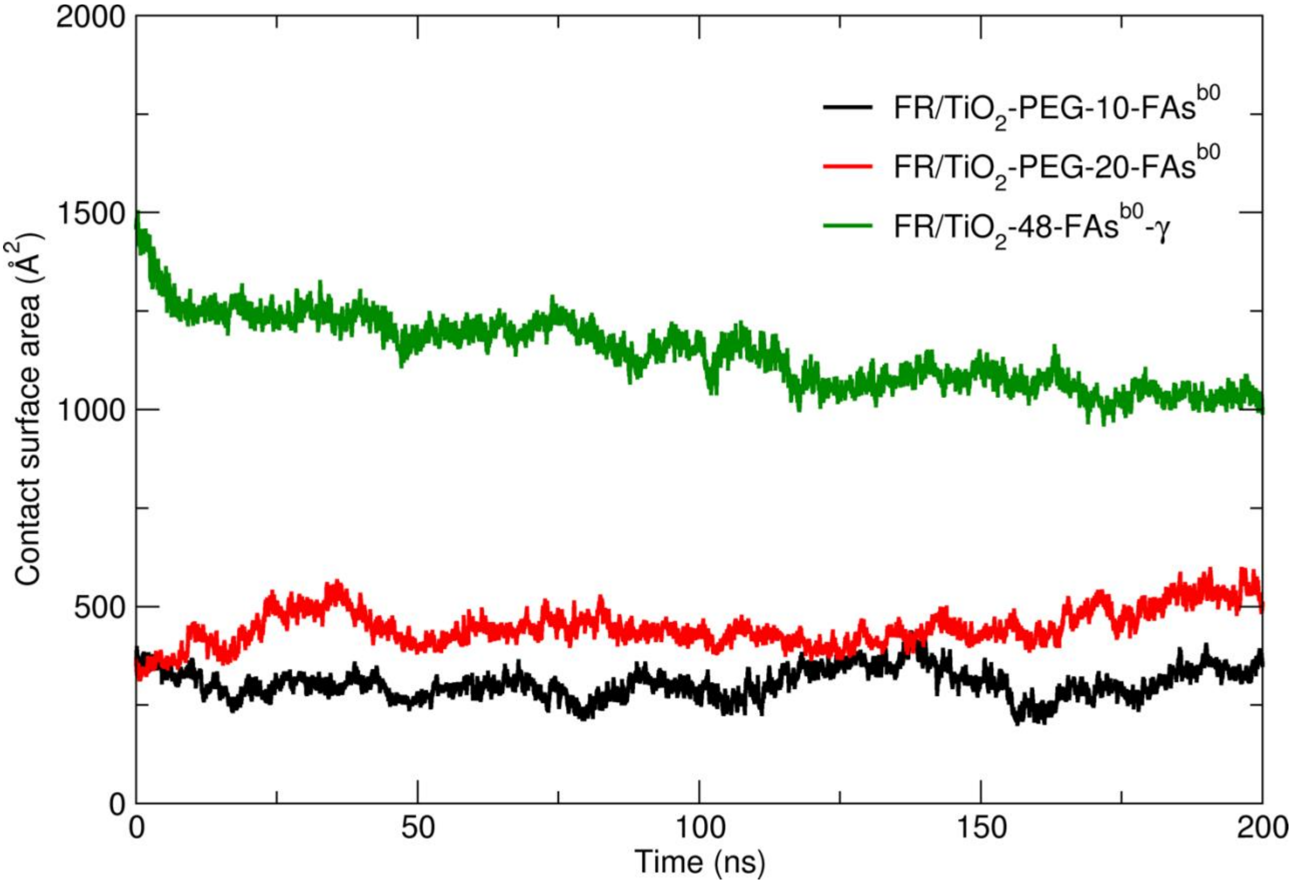

Figure S7. Contact surface area between the functionalized NP and the FR along the 200 ns MD simulations for the FR/TiO_2-PEG-10-FAs^{b0}, FR/TiO_2-PEG-20-FAs^{b0} and FR/TiO_2-48-FAs^{b0}-γ systems.

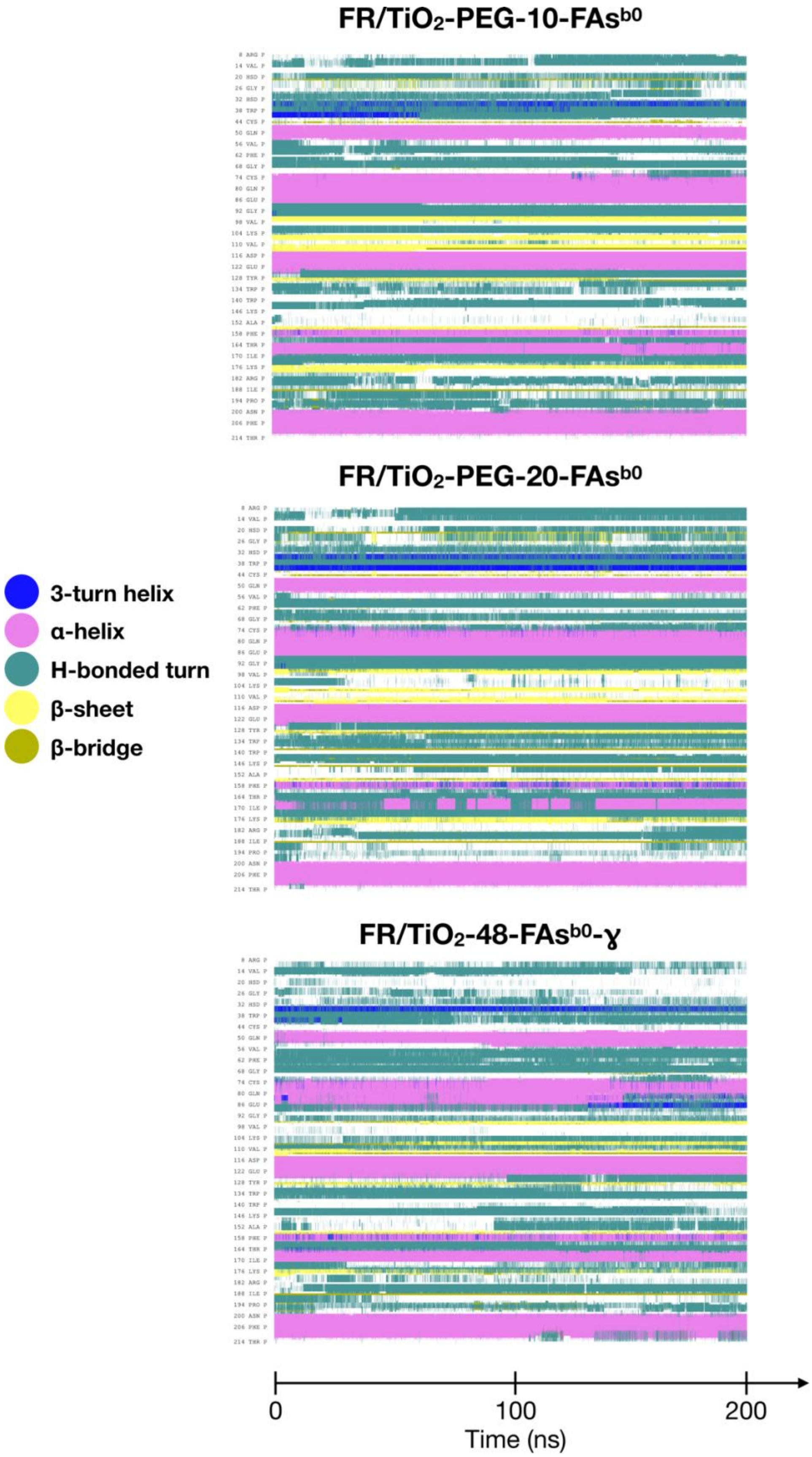

Figure S8. Secondary structure analysis performed along the 200 ns MD simulations of the FR/TiO_2-PEG-10-FAs^{b0}, FR/TiO_2-PEG-20-FAs^{b0} and FR/TiO_2-48-FAs^{b0}-γ systems.

Table S3. Number of intermolecular hydrogen bonds and non-bonded interaction energies along the last 50 ns of the 200 ns MD production simulations of the FR/TiO_2-PEG-10-FAs^{b0}, FR/TiO_2-PEG-20-FAs^{b0} and FR/TiO_2-48-FAs^{b0}-γ systems.

	**H-bonds number**	**Interaction energy (kcal mol^{-1})**	
		**VdW**	**Electrostatic**
	**FR/TiO_2-PEG-10-FAs^{b0}**		
LIG^{b0}-FR	0.5 (± 0.6)	-30 (± 3)	-25 (± 5)
LIG^{b0}-wat	6 (± 2)	-9 (± 4)	-92 (± 10)
FR-wat	253 (± 12)	-503 (± 45)	-5526 (± 356)
FAs^{b0}-FR	0.002 (± 0.04)	-0.5 (± 0.9)	-0.3 (± 10)
FAs^{b0}-TiO_2	-	-	-
FAs^{b0}-FAs^{b0}	0.3 (± 0.7)	-14 (± 3)	-10 (± 7)
FAs^{b0}-wat	50 (± 5)	-148 (± 16)	-996 (± 79)
PEG-FR	0.04 (± 0.2)	-16 (± 4)	-4 (± 5)
LIG^{b0}-Leu59	0.2 (± 0.4)	-2.3 (± 0.8)	-5 (± 2)
LIG^{b0}-Asp81	0.3 (± 0.5)	-0.006 (± 0.8)	-16 (± 3)
LIG^{b0}-Trp102	-	-7 (± 1)	-1.5 (± 0.9)
LIG^{b0}-Arg103	-	-1.0 (± 0.4)	-4 (± 2)
FR-TiO_2+PEG+FAs^{b0}	0.5 (± 0.6)	-47 (± 5)	-29 (± 12)
	**FR/TiO_2-PEG-20-FAs^{b0}**		
LIG^{b0}-FR	1.4 (± 0.6)	-37 (± 4)	-77 (± 7)
LIG^{b0}-wat	3 (± 1)	-9 (± 3)	-47 (± 8)
FR-wat	235 (± 11)	-442 (± 38)	-4996 (± 325)
FAs^{b0}-FR	1.2 (± 0.6)	-12 (± 5)	-36 (± 13)
FAs^{b0}-TiO_2	-	-1.0 (± 0.4)	-2 (± 1)
FAs^{b0}-FAs^{b0}	0.5 (± 0.9)	-68 (± 9)	-30 (± 17)
FAs^{b0}-wat	88 (± 7)	-308 (± 32)	-2066 (± 140)
PEG-FR	0.01 (± 0.09)	-10 (± 3)	0.6 (± 2)
LIG^{b0}-Leu59	0.2 (± 0.4)	-3.2 (± 0.7)	-6 (± 1)
LIG^{b0}-Asp81	0.8 (± 0.4)	1 (± 1)	-20 (± 3)
LIG^{b0}-Trp102	-	-7.8 (± 0.8)	-0.6 (± 0.5)
LIG^{b0}-Arg103	0.6 (± 0.5)	1 (± 2)	-35 (± 4)
FR-TiO_2+PEG+FAs^{b0}	2.6 (± 0.7)	-59 (± 7)	-112 (± 15)
	**FR/TiO_2-48-FAs^{b0}-γ**		
LIG^{b0}-FR	1.3 (± 0.7)	-24 (± 3)	-55 (± 5)
LIG^{b0}-wat	2 (± 1)	-7 (± 2)	-36 (± 6)
FR-wat	238 (± 12)	-449 (± 39)	-5091 (± 340)
FAs^{b0}-FR	3 (± 1)	-94 (± 8)	-123 (± 25)
FAs^{b0}-TiO_2	3.4 (± 0.7)	-345 (± 22)	-191 (± 15)
FAs^{b0}-FAs^{b0}	6 (± 2)	-396 (± 27)	-224 (± 27)
FAs^{b0}-wat	215 (± 11)	-581 (± 47)	-4162 (± 276)
LIG^{b0}-Asp81	0.3 (± 0.5)	0.9 (± 0.5)	-21 (± 2)
LIG^{b0}-Trp102	0.8 (± 0.4)	-10 (± 1)	-11 (± 1)
LIG^{b0}-Arg103	0.02 (± 0.14)	-0.5 (± 0.6)	-7 (± 2)
LIG^{b0}-Arg106	-	-0.08 (± 0.02)	-0.31 (± 0.08)
FR-TiO_2-FAs^{b0}	4 (± 1)	-118 (± 9)	-246 (± 25)

In the FR/TiO_2-PEG-10-FAs^{b0} system the vdW energy is slightly more negative but comparable to the electrostatic energy and the hydrogen bonds number is about a third inferior with respect to the other two systems, where instead electrostatics has a more relevant contribution. In contrast, LIG^{b0} interacts more with water in the FR/TiO_2-PEG-10-FAs^{b0} system. In addition, we register an increase in the FAs^{b0}-FR, FAs^{b0}-TiO_2, FAs^{b0}-FAs^{b0} and FAs^{b0}-water interaction energy as the number of FAs^{b0} increases. More on detail about the interactions between LIG^{b0} and the amino acids in the FR binding pocket, we observe a strong electrostatic interaction with Asp81 in all the three systems, as well as with Arg103, which is more pronounced in the FR/TiO_2-PEG-20-FAs^{b0} system. There is also a notable interaction with Trp102 which is predominantly of a hydrophobic nature for the PEGylated systems, whereas in the FR/TiO_2-48-FAs^{b0}-γ system electrostatic and vdW forces have the same extent. At last, we find a modest interaction, mostly electrostatic, with Leu59 for the PEGylated systems, and a weak interaction with Arg106 for the non-PEGylated system.

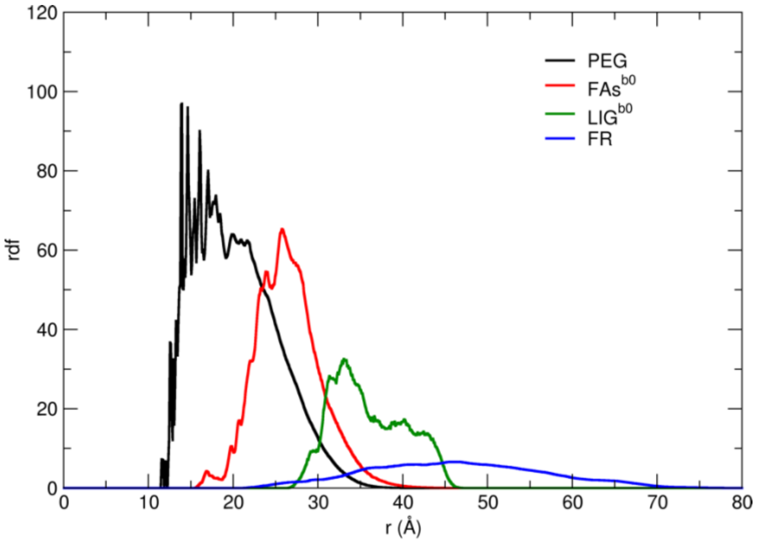

(a)

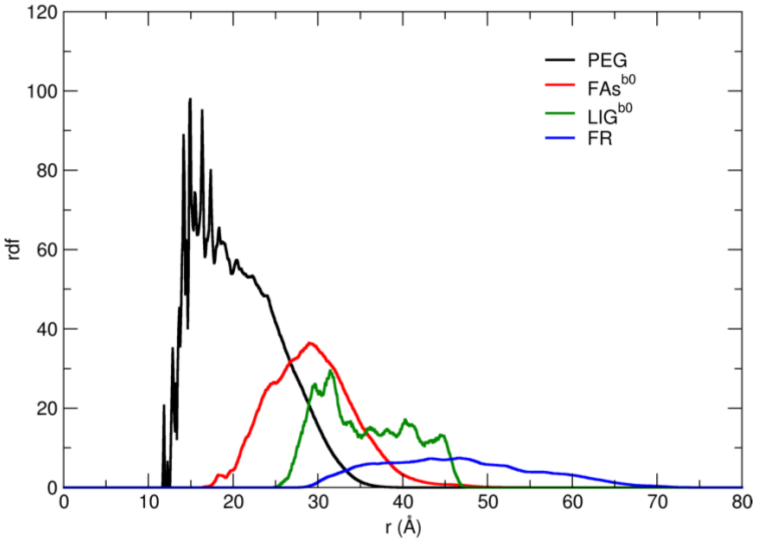

(b)

Figure S9. Radial distribution function profiles of PEG, FAs^{b0}, LIG^{b0} and FR calculated with respect to the Ti atom at the center of the NP, for the FR/TiO_2-PEG-10-FAs^{b0} (a) and FR/TiO_2-PEG-20-FAs^{b0} (b) systems, averaged on the last 50 ns of the 200 ns MD simulation.

We observe that while the PEG plots look very similar for both systems, the FAs^{b0} profile is more shifted towards the bulk-water phase for FR/TiO_2-PEG-20-FAs^{b0} than for FR/TiO_2-PEG-10-FAs^{b0} (29 Å vs 26 Å from the NP center), which indicates a greater exposition of the undocked FAs^{b0} to the solvent and at the same time a more pronounced FR-FAs^{b0} interaction. The other way around is for the folic acid molecule inside the FR binding pocket and for FR itself, as the LIG^{b0} and the FR profiles are found at shorter distances from the NP center in the FR/TiO_2-PEG-20-FAs^{b0} system, suggesting a higher interaction of FR with the PEGylated FAs^{b0}-functionalized nanosystem, in line with the results of the H-bonds and non-bonded interaction energy analysis in **Table S3**.

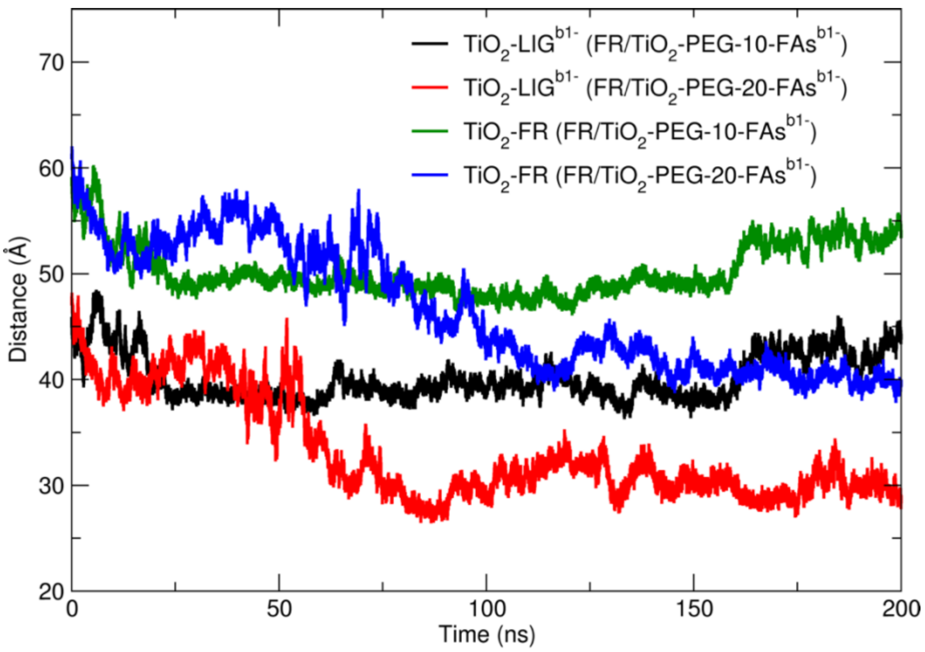

(a)

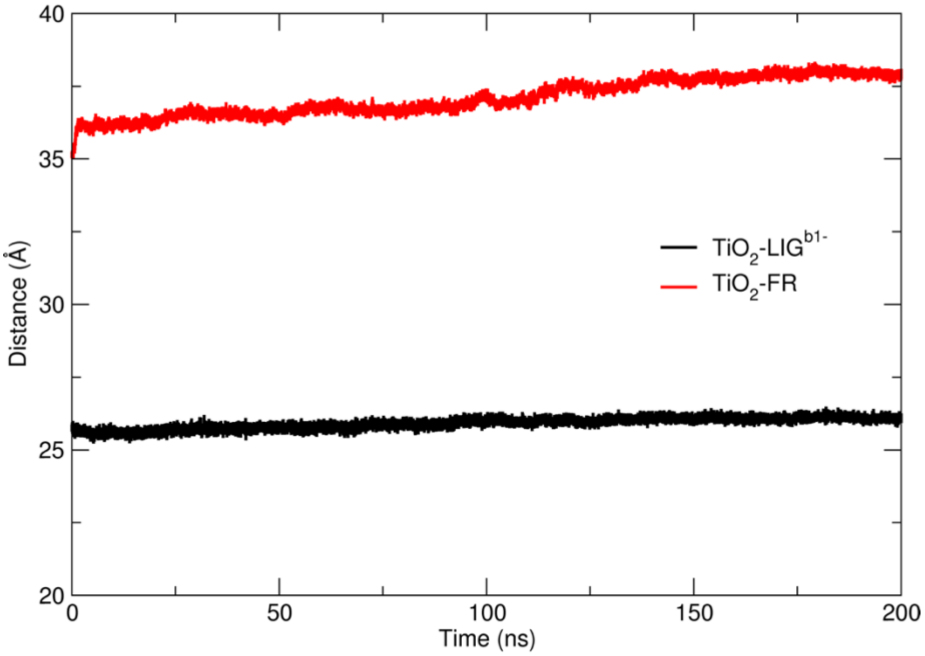

(b)

Figure S10. Time evolution of TiO_2-LIG^{b1-} and TiO_2-FR centers of mass distances along the 200 ns MD simulations for the FR/TiO_2-PEG-10-FAs^{b1-} and the FR/TiO_2-PEG-20-FAs^{b1-} systems (a) and for the FR/TiO_2-48-FAs^{b1-}-γ system (b).

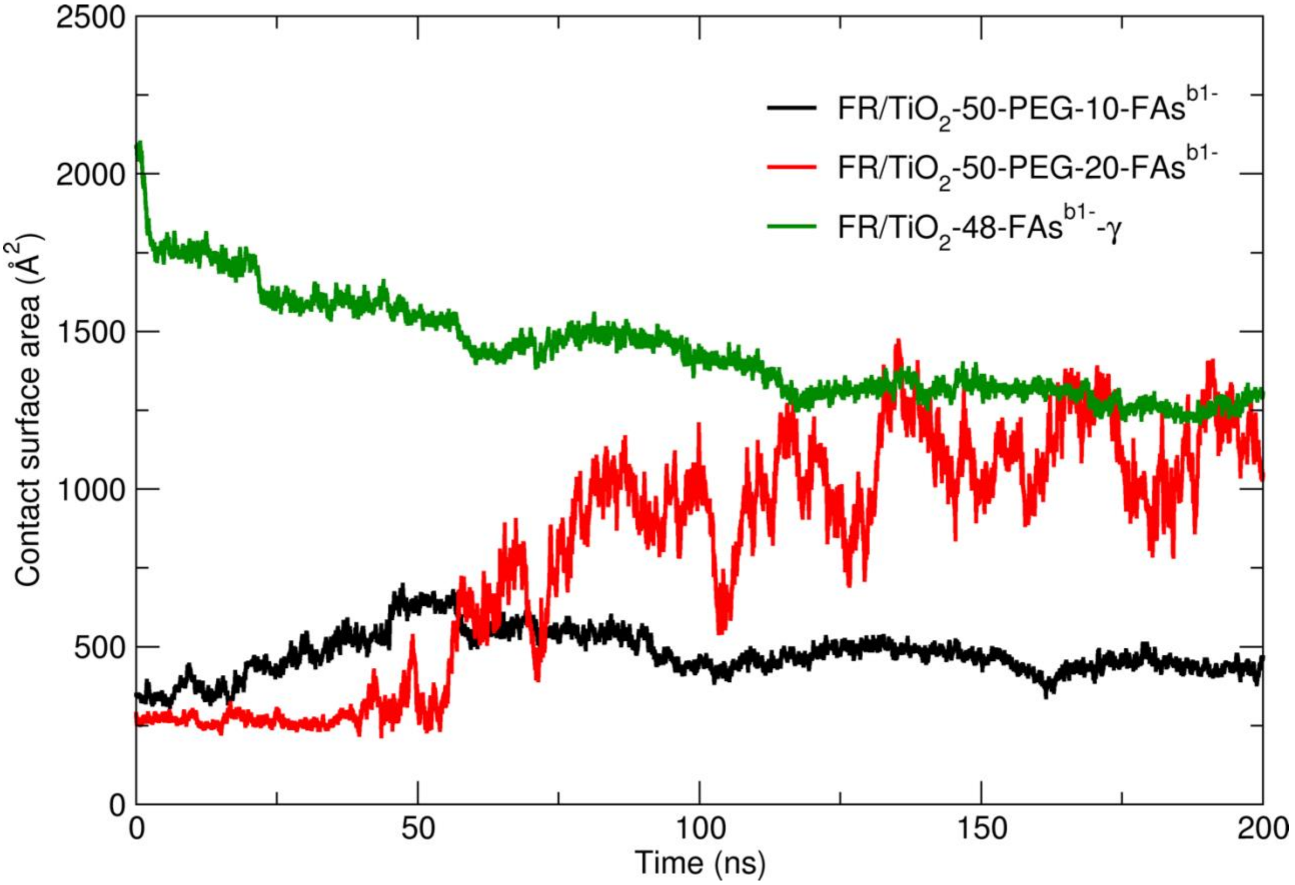

Figure S11. Contact surface between the functionalized NP and the FR along the 200 ns MD simulations for the FR/TiO_2-PEG-10-FAs^{b1-}, FR/TiO_2-PEG-20-FAs^{b1-} and FR/TiO_2-48-FAs^{b1-}-γ systems.

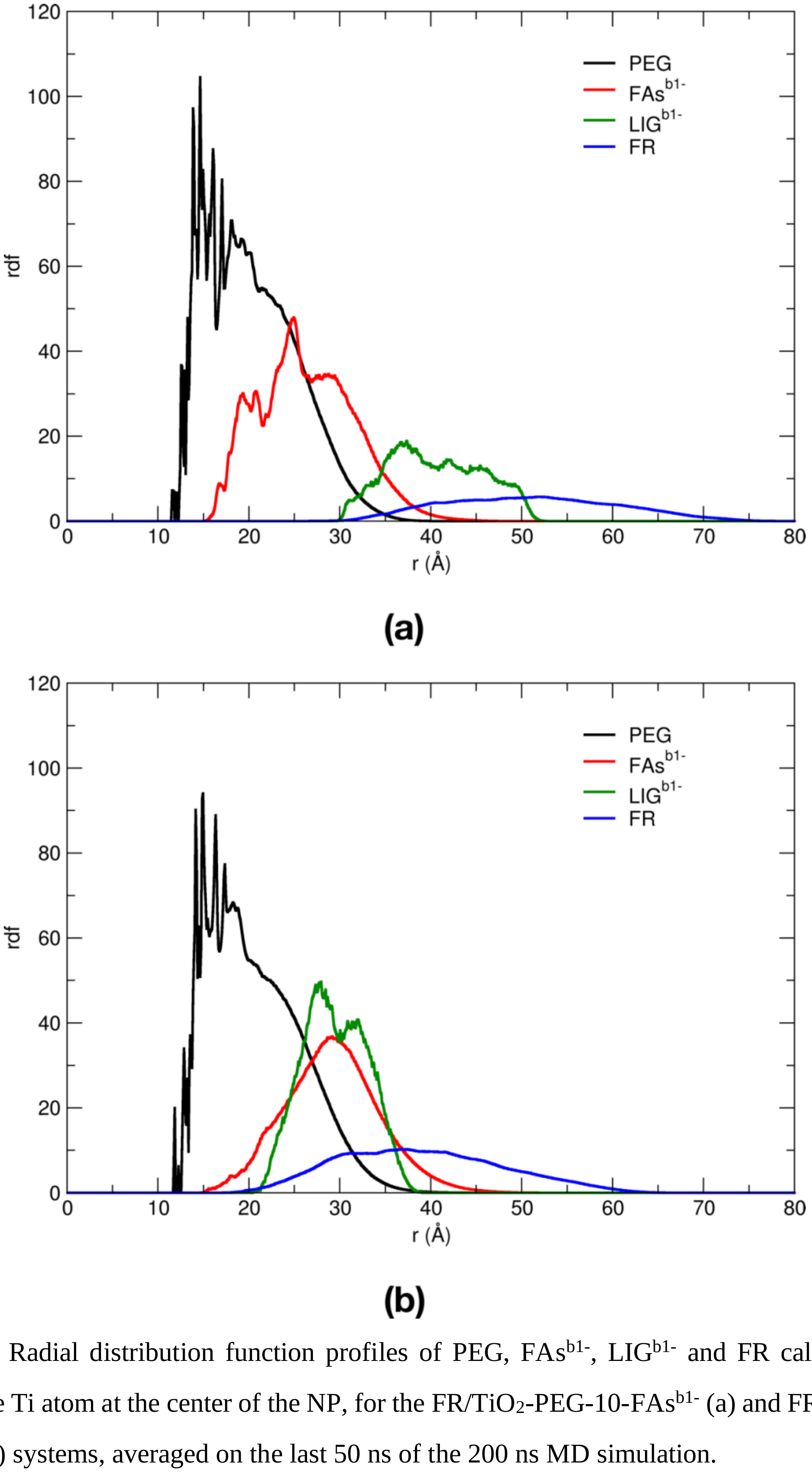

Figure S12. Radial distribution function profiles of PEG, FAs^{b1-}, LIG^{b1-} and FR calculated with respect to the Ti atom at the center of the NP, for the FR/TiO_2-PEG-10-FAs^{b1-} (a) and FR/TiO_2-PEG-20-FAs^{b1-} (b) systems, averaged on the last 50 ns of the 200 ns MD simulation.

Table S4. Number of intermolecular hydrogen bonds and non-bonded interaction energies along the last 50 ns of the 200 ns MD production simulations of the FR/TiO_2-PEG-10-FAs^{b1-}, FR/TiO_2-PEG-20-FAs^{b1-} and FR/TiO_2-48-FAs^{b1-}-γ systems.

	**H-bonds number**	**Interaction energy (kcal mol^{-1})**	
		**VdW**	**Electrostatic**
		**FR/TiO_2-PEG-10-FAs^{b1-}**	
LIG^{b1-}-FR	1.8 (± 0.9)	-29 (± 5)	-100 (± 11)
LIG^{b1-}-wat	6 (± 1)	-0.08 (± 3)	-122 (± 13)
FR-wat	233 (± 12)	-470 (± 38)	-5056 (± 320)
FAs^{b1-}-FR	0.5 (± 0.7)	-7 (± 2)	-22 (± 10)
FAs^{b1-}-TiO_2	-	-1.5 (± 0.6)	-1.4 (± 0.9)
FAs^{b1}—FAs^{b1-}	0.04 (± 0.3)	-12 (± 3)	-4 (± 5)
FAs^{b1-}-wat	78 (± 7)	-86 (± 17)	-1818 (± 121)
PEG-FR	-	-12 (± 3)	-3 (± 2)
LIG^{b1-}-Leu59	0.06 (± 0.2)	-2.8 (± 0.9)	-4 (± 2)
LIG^{b1-}-Asp81	0.5 (± 0.5)	2 (± 1)	-23 (± 3)
LIG^{b1-}-Trp102	-	-5 (± 2)	-0.8 (± 0.9)
LIG^{b1-}-Arg103	0.5 (± 0.5)	0.7 (± 1)	-29 (± 4)
LIG^{b1-}-Arg61	-	-4.9 (± 0.7)	-0.8 (± 0.9)
LIG^{b1-}-Lys19	0.7 (± 0.5)	0.6 (± 2)	-35 (± 8)
LIG^{b1-}-Arg106	0.05 (± 0.2)	-0.3 (± 0.9)	-6 (± 2)
FR/TiO_2+PEG+FAs^{b1-}	2 (± 1)	-48 (± 13)	-125 (± 15)
		**FR/TiO_2-PEG-20-FAs^{b1-}**	
LIG^{b1-}-FR	1.6 (± 0.9)	-26 (± 4)	-65 (± 18)
LIG^{b1-}-wat	5 (± 2)	2 (± 4)	-119 (± 24)
FR-wat	191 (± 12)	-476 (± 45)	-4976 (± 346)
FAs^{b1-}-FR	1 (± 1)	-21 (± 12)	-51 (± 42)
FAs^{b1-}-TiO_2	-	-2 (± 2)	-3 (± 7)
FAs^{b1-}-FAs^{b1-}	0.6 (± 1)	-25 (± 13)	-33 (± 26)
FAs^{b1-}-wat	106 (± 8)	-188 (± 30)	-3514 (± 254)
PEG-FR	1 (± 1)	-59 (± 25)	-43 (± 33)
LIG^{b1-}-Asp81	0.6 (± 0.5)	0.9 (± 1)	-20 (± 4)
LIG^{b1-}-Trp102	0.003 (± 0.05)	-1 (± 1)	-0.2 (± 1)
LIG^{b1-}-Arg103	0.6 (± 0.5)	-0.3 (± 1)	-22 (± 4)
LIG^{b1-}-Trp171	-	-6 (± 1)	-2 (± 1)
LIG^{b1-}-Lys136	0.4 (± 0.5)	-1 (± 2)	-30 (± 14)
LIG^{b1-}-Arg61	-	-0.4 (± 0.2)	-0.2 (± 0.4)
LIG^{b1-}-Arg106	-	-0.4 (± 0.2)	1 (± 1)
FR/TiO_2+PEG+FAs^{b1-}	4 (± 2)	-106 (± 28)	-159 (± 56)
		**FR/TiO_2/48-FAs^{b1-}-γ**	
LIG^{b1-}-FR	1.4 (± 0.9)	-23 (± 3)	-53 (± 6)
LIG^{b1-}-wat	5 (± 1)	-2 (± 2)	-96 (± 10)
FR-wat	227 (± 10)	-463 (± 40)	-4730 (± 338)
FAs^{b1-}-FR	5 (± 2)	-71 (± 7)	-151 (± 15)
FAs^{b1-}-TiO_2	5 (± 1)	-207 (± 16)	-658 (± 47)
FAs^{b1}—FAs^{b1-}	7 (± 3)	-184 (± 15)	-158 (± 45)

FAs^{b1-}-wat	341 (± 13)	-471 (± 43)	-6782 (± 452)
$LIGb^{1-}$-Arg61	0.07 (± 0.3)	-3.7 (± 0.9)	-4 (± 1)
$LIGb^{1-}$-Asp81	0.5 (± 0.5)	0.6 (± 1)	-21 (± 5)
$LIGb^{1-}$-Arg103	1.0 (± 0.6)	1 (± 1)	-31 (± 3)
FR/TiO_2+FAs^{b1-}	6 (± 2)	-94 (± 8)	-204 (± 16)

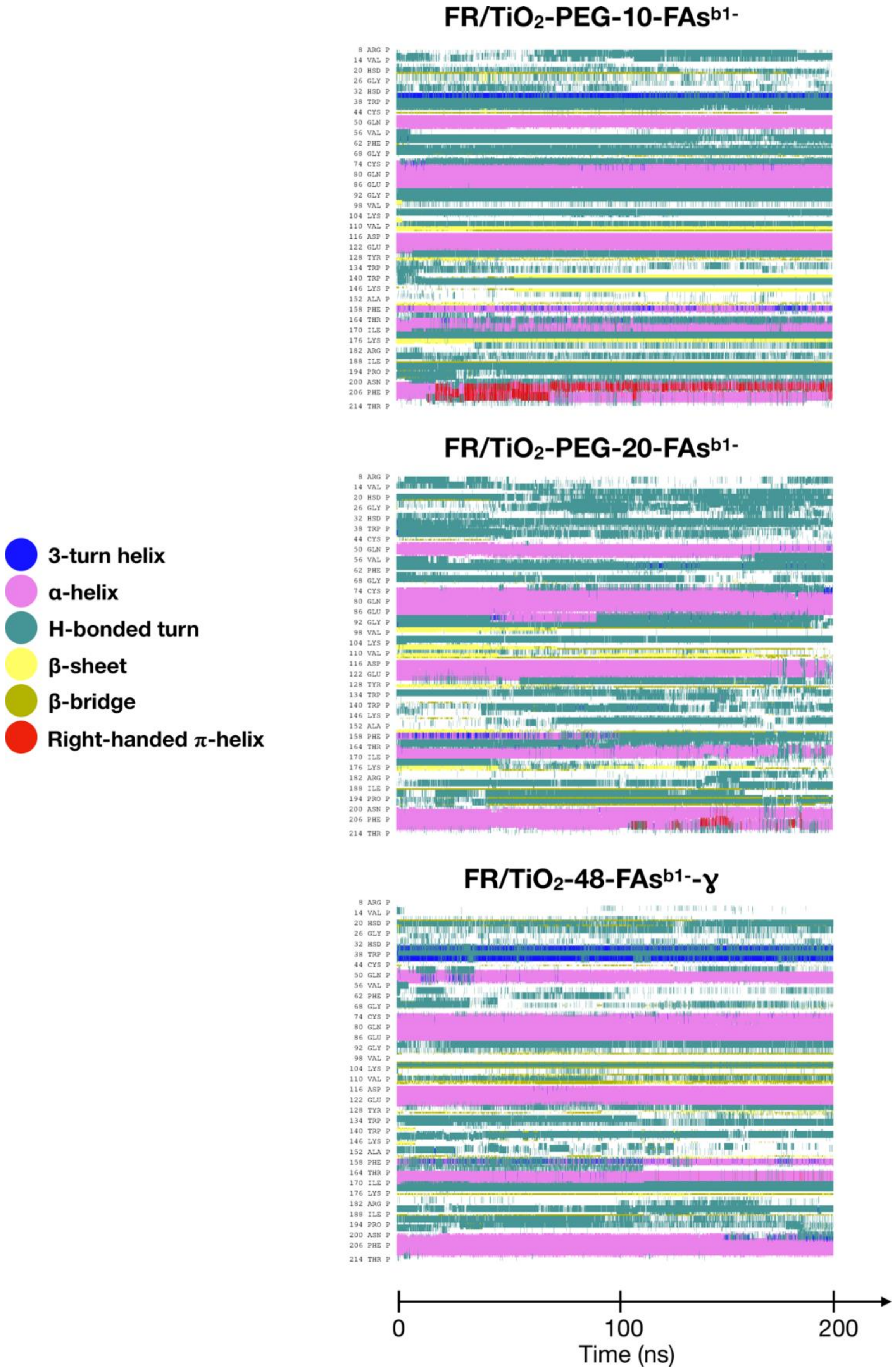

Figure S13. Secondary structure analysis performed along the 200 ns MD simulations of the FR/TiO_2-PEG-10-FAs^{b1-}, FR/TiO_2-PEG-20-FAs^{b1-} and FR/TiO_2-48-FAs^{b1-}-γ systems.

Replicas of MD simulation of FR/TiO_2-PEG-20-FAs^{b1-} system

First, we report the results of the analysis on the replica of the 200 ns MD simulation for the FR/TiO_2-PEG-20-FAs^{b1-} system, where the same equilibrated starting structure of the original simulation was used, but different initial velocities were assigned to the atoms.

In **Figure S14** we report the time evolution along the MD simulation of the distances between the center of mass of the TiO_2 NP and that either of the LIG^{b1-} (TiO_2-LIG^{b1-}) or of the FR (TiO_2-FR).

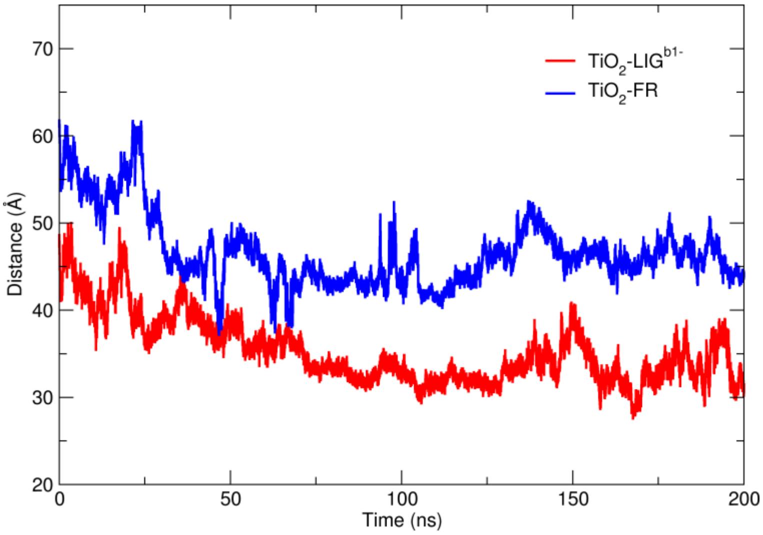

Figure S14. Time evolution of TiO_2-LIG^{b1-} and TiO_2-FR centers of mass distances along the 200 ns MD simulation for the FR/TiO_2-PEG-20-FAs^{b1-} system (first replica).

We observe that both plots are in fair agreement with those of **Figure S10a**, with the TiO_2-LIG^{b1-} and TiO_2-FR distances oscillating around 32 Å (vs. 30 Å) and 45 Å (vs. 40 Å), respectively.

This is also confirmed by the rdf profiles of **Figure S15**, computed along the last 50 ns of the MD simulation.

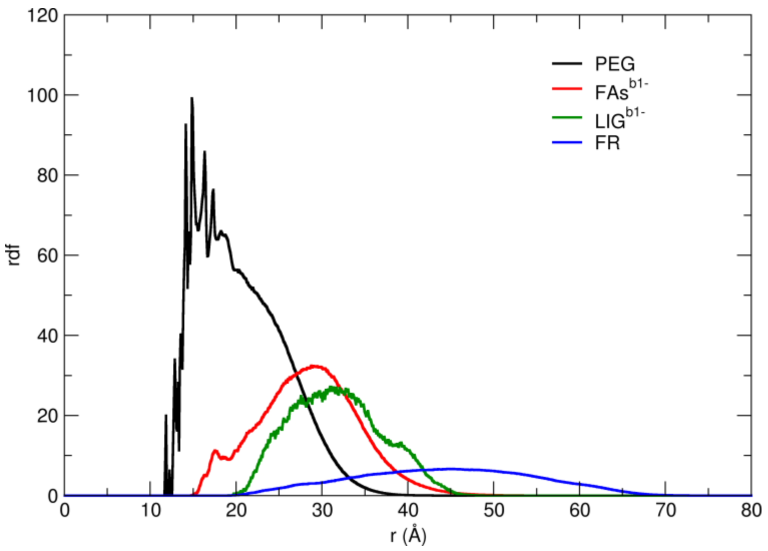

Figure S15. Radial distribution function profiles of PEG, FAs^{b1-}, LIG^{b1-} and FR calculated with respect to the Ti atom at the center of the NP, for the FR/TiO_2-PEG-20-FAs^{b1-} system (first replica), averaged on the last 50 ns of the 200 ns MD simulation.

The slightly higher distance of the protein from the functionalized NP results in a lower contact surface area between the two, as shown in **Figure S16**, where the contact surface area initially increases up to 1000 Å^2 but then settles at about 700 Å^2, to be compared with the value of 1000 Å^2 observed in the first MD run (**Figure S11**).

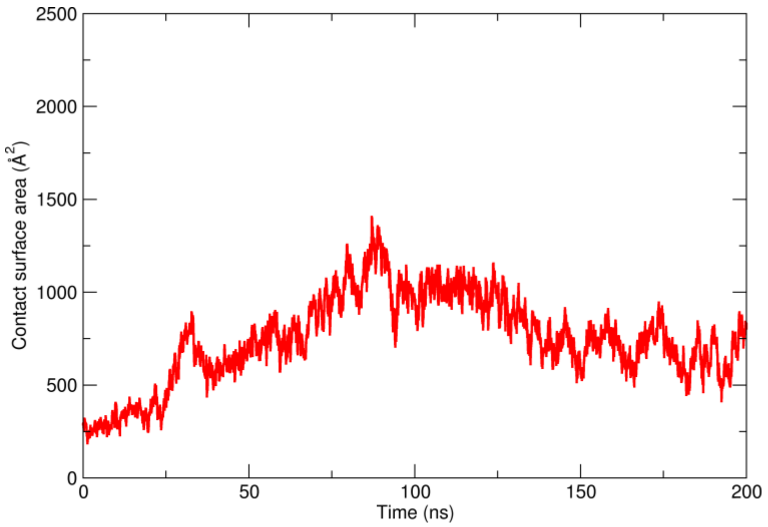

Figure S16. Contact surface area between the functionalized NP and the FR along the 200 ns MD simulation for the FR/TiO_2-PEG-20-FAs^{b1-} system (first replica).

In this replica, LIG^{b1-} is confirmed to be inside the FR binding pocket along all the 200 ns of simulation, based on the LIG^{b1-} distance values from selected AAs of the protein pocket, whose plots are shown in **Figure S17**.

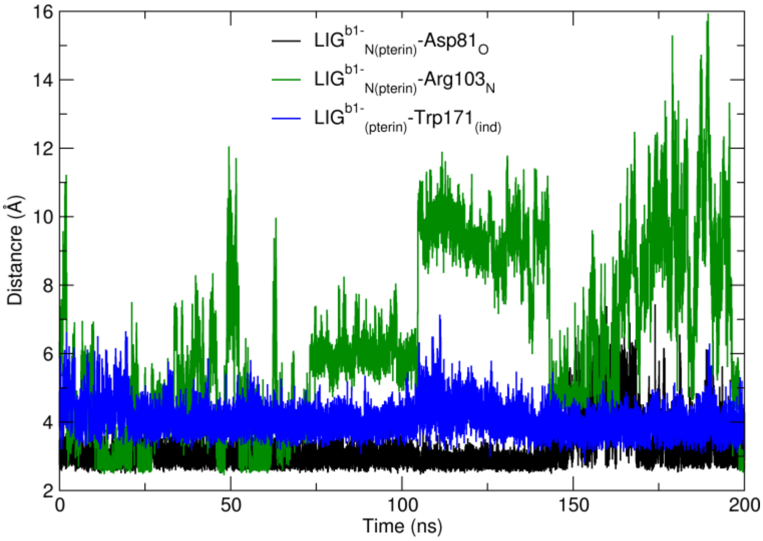

Figure S17. Time evolution of distances between selected atoms of LIG^{b1-} and FR AAs for the 200 ns simulation of the FR/TiO_2-PEG-20-FAs^{b1-} system (first replica) in physiological environment.

As in **Figure 6e**, the ligand is strongly interacting with Asp81 and Trp171 residues by hydrogen bonds and π-π stacking, respectively. The interaction with Arg103 is less constant, similar to what observed in **Figure 6e** for the first run.

Finally, in **Figure S18** we report the FR secondary structure analysis on the 200 ns MD simulation. The results are comparable to those of **Figure S13** of the first simulation, meaning that no change in the protein secondary structure is observed between the two simulations.

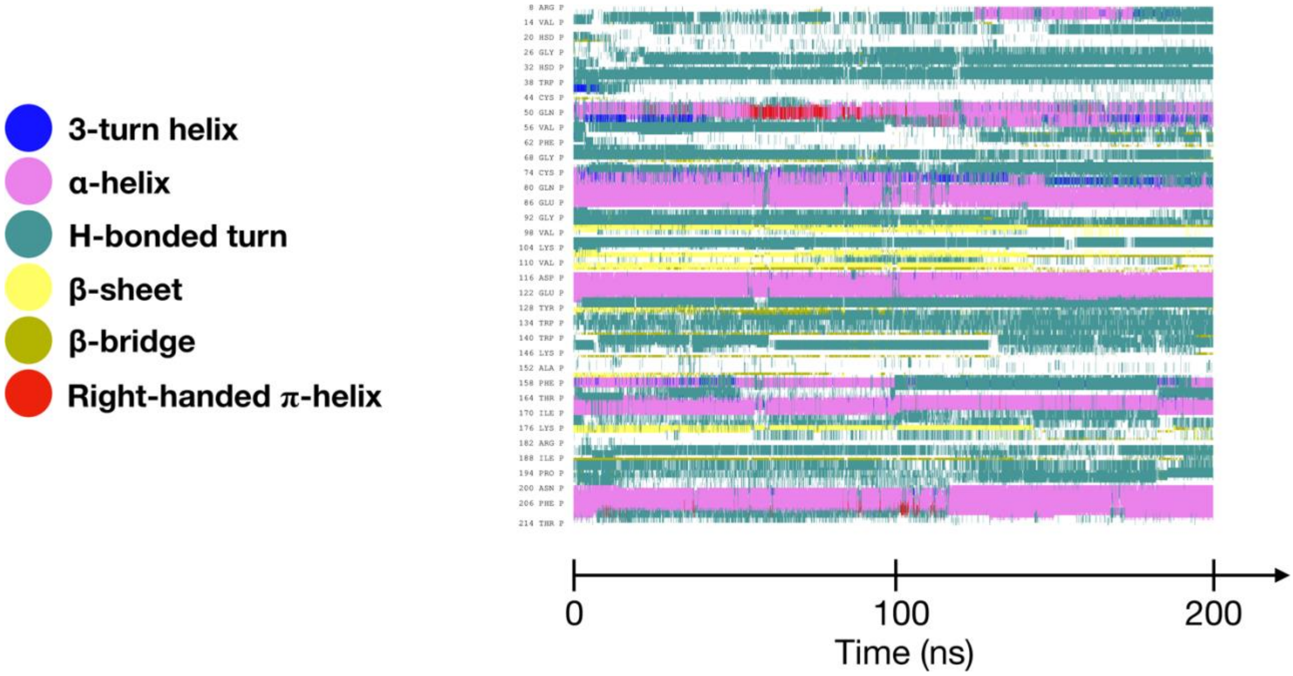

Figure S18. Secondary structure analysis performed along the 200 ns MD simulation of the FR/TiO_2-PEG-20-FAs^{b1-} system (first replica).

In conclusion, replicating the MD run of the FR/TiO_2-PEG-20-FAs^{b1-} system with different initial velocities has demonstrated that the observations on the dynamics of LIG^{b1-} inside the FR binding pocket, on the interaction of the functionalized NP with the protein and on the FR secondary structure are consistent, making our conclusions more robust.

Then, to further support our conclusions, we also report the results of a second replica of 50 ns, starting from the structure at 150 ns of the original simulation of FR/TiO_2-PEG-20-FAs^{b1-} and assigning different initial velocities (**Figures S19-S23**). The analysis presented in **Figures S19-S23** are consistent with those of **Figures 6/S10-13** for the original simulation and **Figures S14-18** for the other simulation discussed above and, therefore, further corroborate the conclusions of this work.

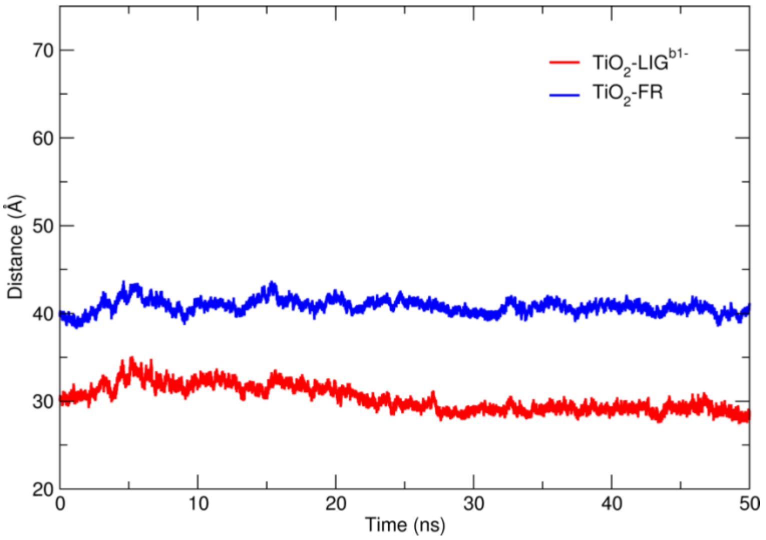

Figure S19. Time evolution of TiO_2-LIG^{b1-} and TiO_2-FR centers of mass distances along the 200 ns MD simulations for the FR/TiO_2-PEG-20-FAs^{b1-} system (second replica).

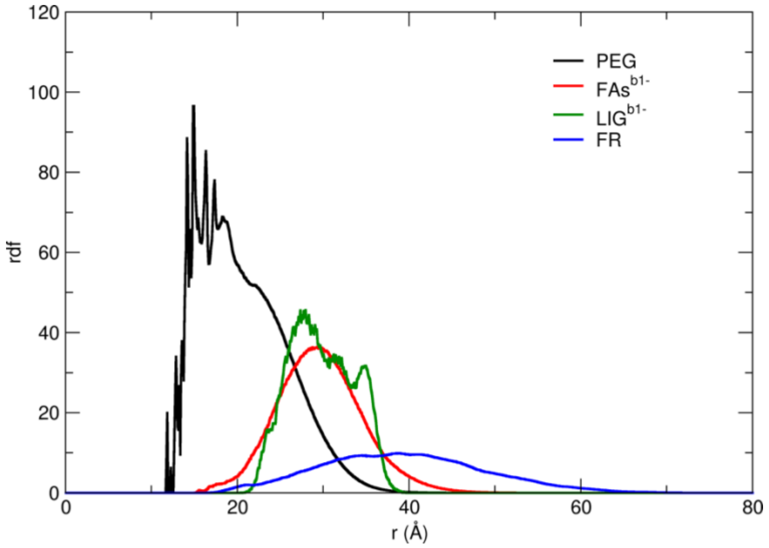

Figure S20. Radial distribution function profiles of PEG, FAs^{b1-}, LIG^{b1-} and FR calculated with respect to the Ti atom at the center of the NP, for the FR/TiO_2-PEG-20-FAs^{b1-} system (second replica), averaged on the last 50 ns of the 200 ns MD simulation.

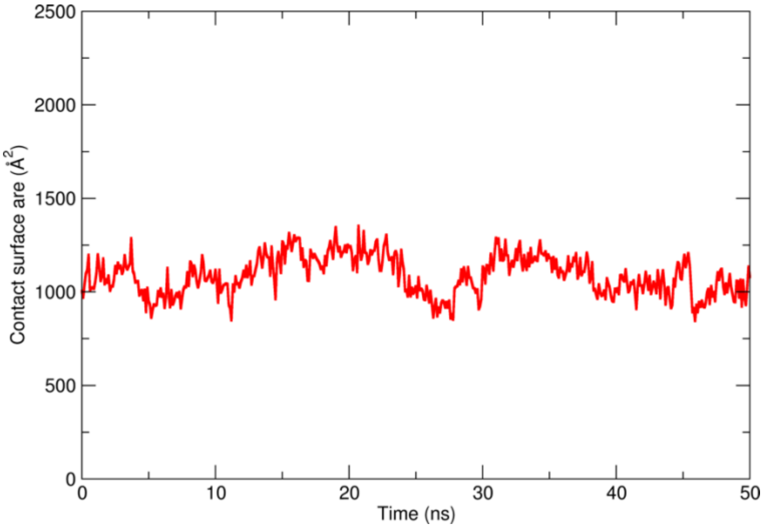

Figure S21. Contact surface area between the functionalized NP and the FR along the 200 ns MD simulations for the FR/TiO_2-PEG-20-FAs^{b1-} system (second replica).

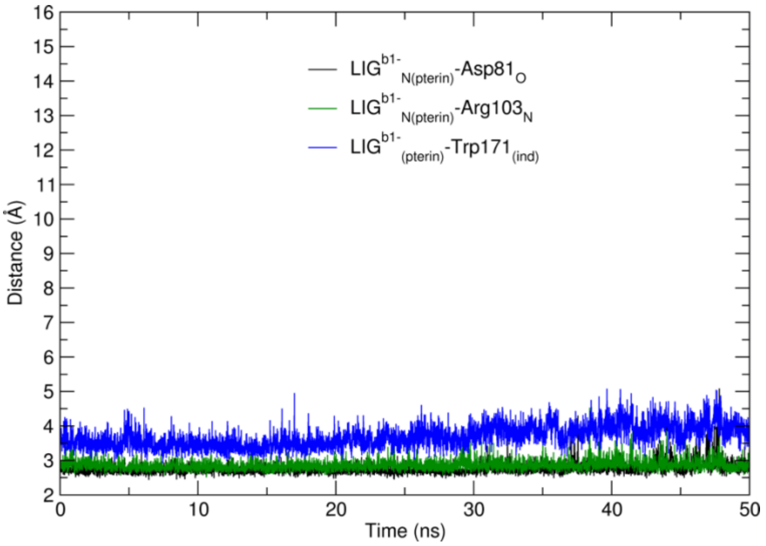

Figure S22. Time evolution of distances between selected atoms of LIG^{b1-} and FR AAs for the 200 ns simulations of the FR/TiO_2-PEG-20-FAs^{b1-} system (second replica) in physiological environment.

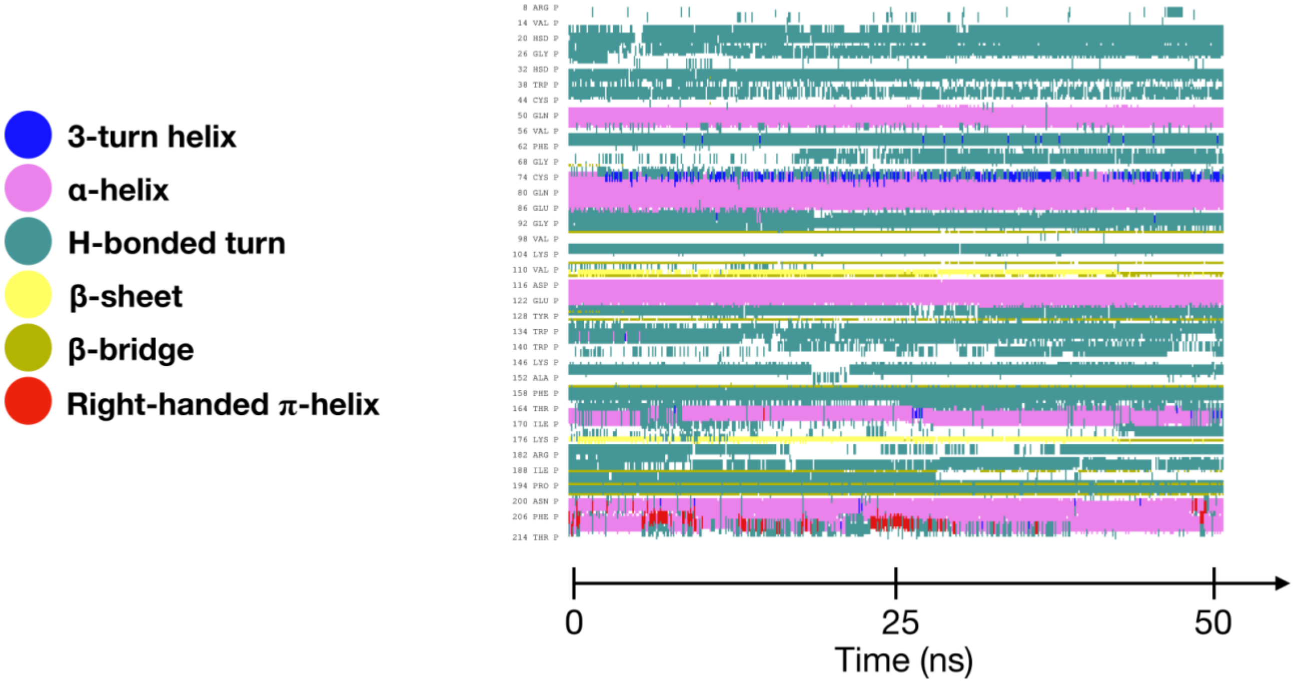

Figure S23. Secondary structure analysis performed along the 200 ns MD simulations of the FR/TiO_2-PEG-20-FAs^{b1-} system (second replica).
